# Application of MXenes in environmental remediation technologies

**DOI:** 10.1186/s40580-021-00255-w

**Published:** 2021-02-17

**Authors:** Mawada Mohammed Tunesi, Razium Ali Soomro, Xi Han, Qizhen Zhu, Yi Wei, Bin Xu

**Affiliations:** 1 State Key Laboratory of Organic-Inorganic Composites, Beijing Key Laboratory of Electrochemical Process and Technology for Materials, Beijing University of Chemical Technology, Beijing, 100029 China; 2 Beijing Advanced Innovation Centre for Soft Matter Science and Engineering, Beijing University of Chemical Technology, Beijing, 100029 China

**Keywords:** MXenes, Environment, Adsorbents, Membrane separation, Photocatalysis, Sensors

## Abstract

MXenes have recently been recognized as potential materials based on their unique physical and chemical characteristics. The widely growing family of MXenes is rapidly expanding their application domains since their first usage as energy materials was reported in 2011. The inherent chemical nature, high hydrophilicity, and robust electrochemistry regard MXenes as a promising avenue for environment-remediation technologies such as adsorption, membrane separation, photocatalysis and the electrocatalytic sensor designed for pollutant detection. As the performance of MXenes in these technologies is on a continuous path to improvement, this review intends to cumulatively discuss the diversity and chemical abilities of MXenes and their hybrid composites in the fields mentioned above with a focus on MXenes improving surface-characteristics. The review is expected to promote the diversity of MXenes and their hybrid configuration for advanced technologies widely applied for environmental remediation.

## Introduction

The rapid advancement in global industrialization has raised severe environmental concerns [1, 2]. Here, the release of industrial waste effluent without proper treatment is one of the primary causes of environmental pollution. The large volume of contaminated effluent contains a mixture of toxic azo dyes, pesticides, and heavy metals from various industries related to coloring, printing, plastics, leather, food, and pharmaceuticals [3, 4]. In general, environmental pollutants could be classified as organic and inorganic, where toxic heavy metals and dyes are the primary sources of water contamination. The pharmaceutical residues and chemical toxins are another emerging class of environmental pollutants, which have shown deteriorating behaviour towards human life. Thus, removing such contaminants using a safe, chemically practical, and environmentally friendly approach is a priority. Among the prominent technologies, adsorption [5], membrane separation [6], photocatalysis [7], and the electrocatalytic sensor systems [8], designed mainly for capturing, isolating, degrading, and detecting the environmental pollutants/toxins, are considered as practical and economically viable remediation technologies.

To date, a variety of materials such as polymers, carbon-based materials, metals, and metal oxides have been considered for developing robust adsorbent, photocatalyst, or sensing platform for the technologies mentioned above [9, 10]. In this context, MXenes, a new family of transition metal carbides/nitrides, have gained significant scientific attention based on their unique 2D surface characteristics, high hydrophilicity, and surface-functionalization ability. Since the first report on MXenes, i.e., Naguib et al., more than 30 different MXene members have been successfully synthesized and reported for their intrinsic characteristics [11, 12]. In general, MXenes have a representative formula of M_*n*+*1*_X_*n*_T_*x*_, where "M" denotes a transition metal, "X" can be a carbon (C) or nitride (N), *n* = 1∼3, and T_*x*_ represents the associated surface functionality (like –OH, –F, –O) [13–15]. These surface functionalities have strong relevance to the physical and chemical properties of MXene materials, which further influence their potential in surface-interactive applications such as adsorption or membrane separation. Unlike graphene with low hydrophilicity, MXenes offer high hydrophilicity and activity towards ion-exchange and redox process due to their surface functionalities. Furthermore, the 2D structure, coupled with robust redox ability, have recognized MXenes’ potential in engineering photocatalyst and electrocatalytic sensors for the degradation and the detection of environmental contaminants [16–18]. The rapid development of MXenes in the environmental remediation and detection technologies calls for an objective understanding of the influence of their surface-related properties on the final performance of MXene-based hybrids.

**Fig. 1:**
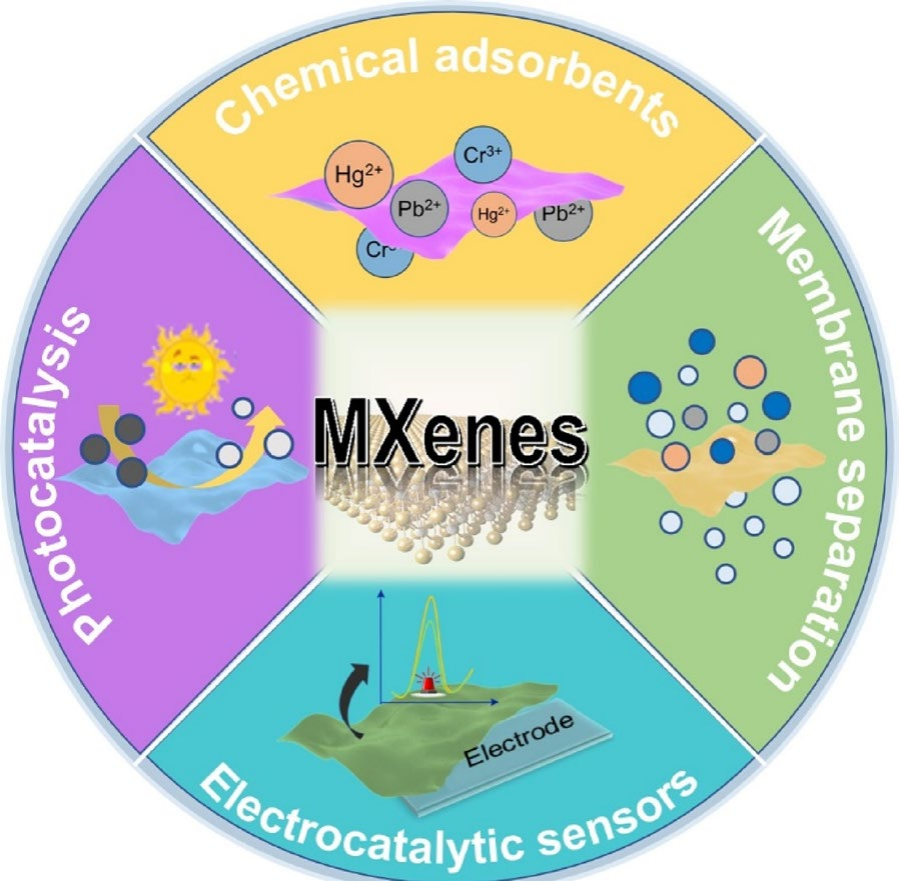
Schematic illustrating the use of MXenes in environmental remediation technologies such as adsorption, membrane separation, photocatalysis, and electrocatalytic sensors

This review discusses the growing fame of MXenes and their hybrids in environmental remediation application, starting with adsorption and membrane separation extending to photocatalysis, with the last section attributed to MXene-based electrocatalytic sensors designed particularly for the identification and quantification of toxins. Figure 1 shows a doughnut chart generalizing the wide-spectrum application of MXenes in these technologies. The discussion is based on the MXenes’ decisive surface characteristics as pristine or in a composite configuration, with the last section dedicated to the conclusion and future-perspective of MXenes in environmental remediation technologies.

## MXenes in chemical adsorption of pollutants

### Chemical adsorbents of dyes

Dyes, which are essential components in textile, paper, and printing industries, despite their practical usage, poses a severe threat to the ecosystem and causes water contamination. Thus, the removal of these dyes is an essential and urgent demand when it comes to environmental remediation. To date, numerous methods have been developed to remove such dyes, and among them, adsorption is the most promising route based on its low cost and preparative applicability. The high specific surface area, negatively charged surface, layered structure, high hydrophilicity have recognized MXenes as ideal adsorbent materials to remove dyes.

**Fig. 2:**
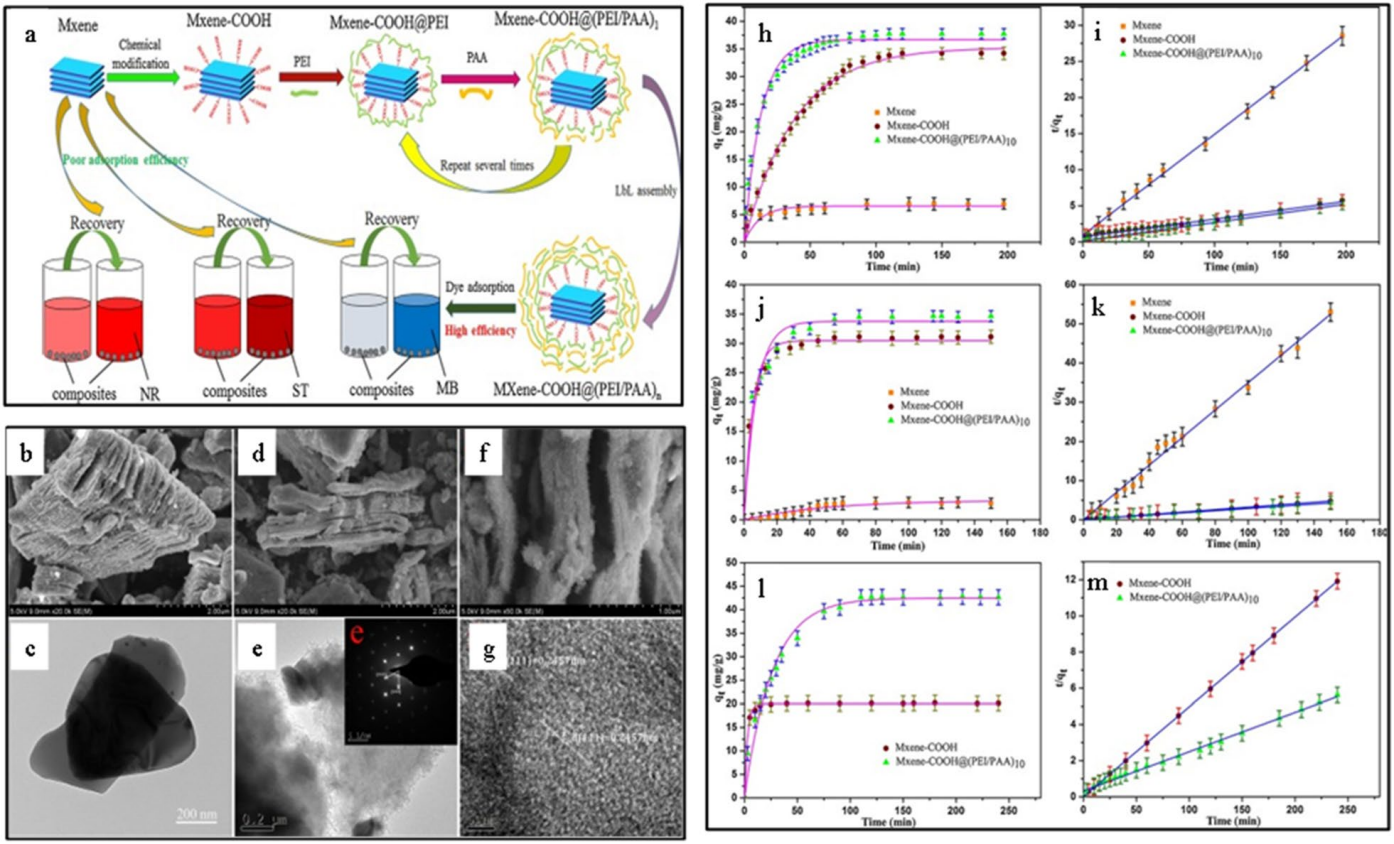
**a** Schematic illustration of the fabrication of the MXene-COOH@(PEI/PAA)_n_ composite. **b**–**g** SEM and TEM images of MXene, MXene-COOH, and the composite. **h**–**m** Adsorption capacities and rate constants for MXene, MXene-COOH, and the composite for MB, ST, and NR dyes at 298 K respectively. Reprint with permission[21]: copyright 2018, Elsevier B.V

The efficient adsorption cationic dyes, such as methylene blue (MB), by multilayered MXene, i.e., ML-Ti_3_C_2_T_*x*,_ were first reported by Mashtalir’s group [19]. The report described the adsorptive properties of ML-Ti_3_C_2_T_*x*_ MXene against a cationic and an anionic dye, i.e., MB and acid blue 80 (AB80), respectively. The results revealed that MB could irreversibly be bonded to Ti_3_C_2_T_*x*_ (with an adsorption capacity of ∼39 mg g^−1^), while AB80 showed negligible adsorption capability towards MXene. The preferential adsorption of the cationic dye over anionic dye was attributed to the electrostatic interactions between the positively charged dye and the negatively charged MXene surface. The adsorption behaviour of dyes over alkali-treated MXenes was later studied by Wei et al. [20]. The authors utilized a facile approach to expand the interlayer spacing and tune the surface functional groups of Ti_3_C_2_T_*x*_ using hot alkaline solution treatment. The interlayer spacing of Ti_3_C_2_T_*x*_ MXene was expanded 29%, and all the –F surface functional groups were transformed to –OH. The alkali treatment enhanced the adsorption capability and accelerated the removal rate of MB dye. Among the treated MXenes, LiOH-Ti_3_C_2_T_*x*_ and NaOH-Ti_3_C_2_T_*x*_ demonstrated the fastest absorption of MB with NaOH-Ti_3_C_2_T_*x*_ achieving the highest adsorption capacity of 189 mg g^−1^. The high adsorption capability was ascribed to the synergism of surface functionalization and enlarged inter-layer adsorption with Langmuir as the standard adsorption model. Moreover, Li et al. [21] reported the chemical modification of MXene with polymers for dye adsorption. Here, MXene-based core–shell composite denoted as MXene-COOH@(PEI/PAA)_n_, was prepared from chemically-modified MXene using a layer-by-layer (LbL) self-assembly approach, as shown in Fig. 2a. The pristine MXene was firstly treated with ClCH_2_COOH to form carboxyl-modified MXene material (MXene-COOH). Then, the MXene-COOH was subjected to polyethene polyimide (PEI) and poly(acrylic acid) (PAA) in a stepwise manner to create a bilayer polymer overcoat around the MXene-COOH via a self-assembly process under electrostatic interactions. The SEM and TEM images presented in Fig. 2b–g indicate the formation of an overcoat-like layer on MXene after polymer’s coupling. The MXene-COOH@(PEI/PAA)_n_ with abundant chemical moieties and hierarchical core-cell structure allowed efficient adsorption of three kinds of dyes, i.e., safranine T (ST, 33.76 mg g^−1^), neutral red (NR, 42.50 mg g^−1^), and MB (36.69 mg g^−1^) (Fig. 2h–m).

In another study, Peng et al. [22] developed a new approach to synthesize 2D MXenes, including Ti_3_C_2_ and Nb_2_C, via the solvothermal treatment of sodium tetrafluoroborate (NaBF_4_) and hydrochloric acid. Here, NaBF_4_ reacted with HCl, resulting in an *in-situ* production of HF, which enabled etching of the MAX phase precursors, i.e., Ti_3_AlC_2_ and Nb_2_AlC. The produced MXenes (h-Ti_3_C_2_ and h-Nb_2_C) possessed superior specific surface area to those prepared using the traditional HF etching method. The h-Ti_3_C_2_ achieved a high MB adsorption capacity of 189 mg g^−1^, whereas h-Nb_2_C showed relatively weaker adsorption towards MB and no adsorption capacity towards methylene orange (MO). It could be explained based on Nb larger atomic mass (92.9) compared to Ti (47.8). Cai et al. [23] transformed 2D Ti_3_C_2_T_*x*_-MXene into the rod-like architecture by modifying the pristine MXene with phytic acid (PA) under hydrothermal conditions. In this case, PA was coupled with the MXene to improve the overall amphiphilicity based on its surface interactive groups, and the hydrothermal reaction time defined the rod-like morphology of the MXene. The PA-MXene composite exhibited enhanced adsorption properties for MB and Rhodamine B (RhB) dyes, with an adsorption capacity of 42 and 22 mg. g^−1^ with 85 and 84% capacity retention after continuous 12 cycles of adsorption, respectively. The use of MXenes in the removal of colored dyes is an area of growing interest. However, there is still room to explore, particularly in the selective removal of pigments based on the surface charge engineering of MXenes.

### Chemical adsorbent of toxic ions

Heavy metal ions are the most dangerous environmental pollutants due to their non-degradable and persistent nature. The long-lasting toxicities of heavy metal ions are effective even at low concentrations, making them high-risk pollutants that require immediate attention. The presently employed techniques that are considered efficient in removing such metal ions include chemical precipitation, filtration, adsorption, and electro-dialysis [24]. In comparison to dyes, whose concentration is relatively high in aquatic systems, removing metal ions is challenging due to their low concentrations (ppm to ppb). However, the methods based on biological processes or chemical reactions are usually ineffective at low concentrations. Thus, the engineered adsorbent needs to be cost-effective and efficient enough to capture toxic metal ions at the ppb level.

MXenes and their derivatives with abundant surface functionalities and large surface areas are considered as potential adsorbents for heavy metal cations. Several reports indicate MXenes have strong adsorption towards various heavy metal ions. Peng et al. [25] developed a functional 2D Ti_3_C_2_T_*x*_-MXene material by an exfoliation and alkalization-intercalation method for Pb ions adsorption. The obtained MXene exhibited a preferential Pb (II) sorption behaviour in the presence of competing cations such as Ca (II)/Mg (II) at high concentrations. The kinetics data confirmed that the sorption equilibrium was achieved in a short duration of 120 s. The MXene could achieve an efficient Pb (II) uptake with a sorption capacity of 4500 kg water per alk-MXene. The experimental and computational modelling confirmed the sorption behaviour to primarily rely on the -OH groups and the activated Ti sites in the MXene, which facilitated the Pb (II) ion-exchange.

**Fig. 3:**
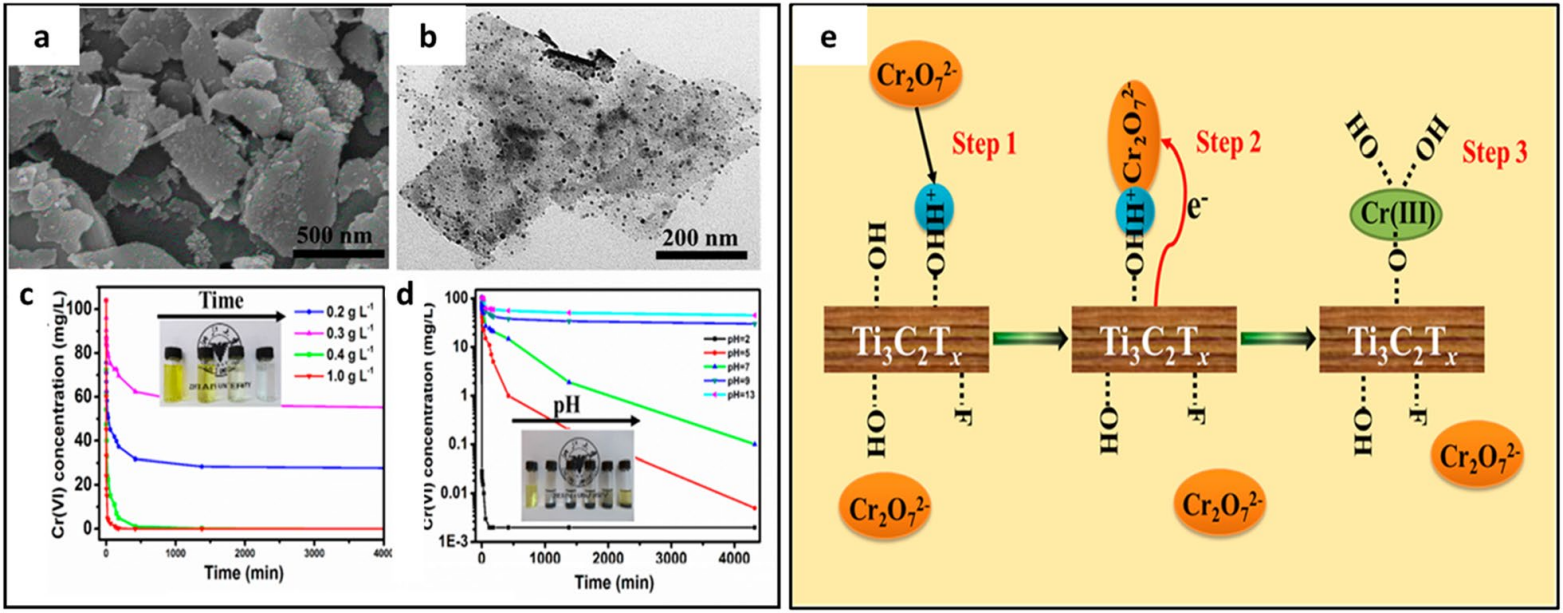
**a** SEM, **b** TEM, **c** dosage effect of the Ti_3_C_2_T_x_ on Cr (VI) removal with insert showing corresponding photo with change in color visible in in the case of 0.4 g; **d** Influence of pH on the Cr (VI) removal efficiency of MXenes; **e** schematic of the charge-interaction based mechanism for the removal of Cr(VI) using Ti_3_C_2_T_*x*_ nanosheets. Reprint with permission [26]: copyright 2015, American Chemical Society

In regard to water contaminants, the chromium ion Cr (VI) is another toxic and carcinogenic ion. Ying et al. [26] utilized 2D Ti_3_C_2_T_*x*_ nanosheets to remove toxic Cr(VI) from water. The complete exfoliation with suitable sheet morphology of the MXene was achieved with 10% HF etchant (Fig. 3a, b). In this case, the Ti_3_C_2_T_*x*_ nanosheets were efficient in removing Cr (VI) with an adsorption capacity of 250 mg g^–1^. Figure 3c, d shows that the Cr (VI) removal efficiency relied directly on the content of MXene and pH of the system, whereas the adsorption was based on the charge-interactions between the positively charge chromium and MXene, as depicted in Fig. 3e. The Cr (VI) residual concentration in the treated water effluent was less than 5 ppb, which was relatively low compared to the standard value of 0.05 ppm.

MXene derivatives can also be used as an adsorbent to the Zou et al. [27] reported an MXene-derived urchin-like rutile TiO_2_-C (u-RTC) composite for Cr(VI) removal. In this case, in situ phase transformation of MXene was carried under FeCl_3_ conditions. First, a layered anatase TiO_2_–C (l-ATC) nanocomposite with dense (001) facets forms, which then transforms to u-RTC subsequent to Fe (III) ion induction. The MXene-derived u-RTC achieved a high Cr (VI) adsorption capacity of ∼225 mg g^−1^, which was much greater than the pristine MXene (∼62 mg g^−1^) and its l-ATC precursor (∼11 mg g^−1^). The first-principles calculations revealed that the bridging oxo-groups within the hybrid system could effectively inhibit the adsorption of H_2_O molecules, thus leading to a high Cr (VI) adsorption capacity. Fard et al. [28] reported the use of MXene for the adsorption of barium ions. In this case, pristine MXene nanosheets demonstrated extraordinary efficiency in removing barium ions with a capacity of 9.3 mg g^−1^ with a removal efficiency reaching up to 100% under the optimum conditions.

The performance of 2D Ti_3_C_2_T_*x*_ MXene nanosheets for the adsorptive removal of copper ions from aqueous media was also investigated [29]. The delaminated-Ti_3_C_2_T_*x*_ showed a high capability to adsorb Cu^2+^ based on its 2D-layer configuration, large specific surface area, and hydrophilicity. Here, the oxygenated moieties on MXenes were responsible for the reductive adsorption of Cu^2+^. Moreover, the Ti_3_C_2_T_*x*_ exhibited a higher and faster Cu^2+^ uptake than the multilayer-Ti_3_C_2_T_*x*,_ reflecting the importance of layer thickness. The maximum adsorption capacity, in this case, was 78.45 mg g^–1^, where 80% of the total Cu ions were adsorbed within 1 min. In terms of commercial competitiveness, the adsorption capacity of the delaminated-Ti_3_C_2_T_*x*_ was 2.7 times higher than activated carbon. Shahzad et al. [30] used a Fe_2_O_3_/MXene nanocomposite for the adsorption of mercuric ions (Hg(II)). The nanocomposite removed the Hg (II) in a wide pH range with an exceptional uptake capacity of 1128.41 mg g^−1^. The adsorption behaviour was well-fitted to Redlich-Peterson adsorption isotherm, with adsorption behaviour following pseudo-second-order kinetics.

MXenes, based on their surface-active groups, have also been considered effective against other toxic ions. Among many, the excessive discharge of phosphates into the aqueous system could result in eutrophication. In this context, Zhang et al. [31] prepared a sandwiched structural MXene-iron oxide (MXI) composite by the intercalation of magnetic ferric oxide into MXene. The MXI composite exhibited remarkable applicability for trace phosphate sequestration. Compared to the commercial adsorbents, the MXI composite showed a fast separation of 120 s, together with the superior treatment capacities of 2100 and 2400 kg g^−1^ in simulated and real phosphate wastewater, respectively. The efficient sequestration was ascribed to the formation of a unique nano-ferric oxide morphology, where ultrafine nano-Fe_2_O_3_ particles could intercalate into the interior layers of MXene, enlarging the interlayer distance and stimulating the available overlapping activated layers. Pandey et al. [32] utilized Ti_3_C_2_T_*x*_ MXene in chemical reduction and removal of bromate ions (BrO^3−^), which are toxic if present in drinking water. Its Ti–C active layer could reduce bromate to bromide while transforming to TiO_2_ nanocrystals. The reduction performance of the Ti_3_C_2_T_*x*_ nanosheets was affected by the concentration of MXene, contact time, pH, and temperature of the system. This system allowed excellent bromate uptake (∼321.8 mg g^−1^) within 50 min at pH 7 and 25 °C.

The specific uptake capacities of MXenes and MXene-based composites and derivatives towards dyes and ionic pollutants are listed in Table 1. Although the MXene-based materials are promising candidates for removing the toxic ions, there are still major challenges, such as the restacking of MXene layers and selective adsorption of toxic ions from a complex matrix require immediate attention before such material could be anticipated as commercial sorbents.

**Table 1:** Summary of specific uptake capacities of MXenes and MXene-based composites for dyes and ionic pollutants

MXene or derivative	Pollutant	Uptake capacity (mg/g)	Reference
Ti_3_C_2_T_*x*_	MB	100	[20]
LiOH-Ti_3_C_2_T_*x*_		121	
NaOH-Ti_3_C_2_T_*x*_		189	
KOH-Ti_3_C_2_T_*x*_		77	
MXene /COOH@(PEI/PAA)	ST	33.76	[21]
	NR	42.50	
	MB	36.69	
h-Ti_3_C_2_	MB	24	[22]
	MO	–	
Phytic Acid-MXene	MB	42	[23]
	RhB	22	
Ti_3_C_2_(OH/ONa)_*x*_F_*2–x*_	Pb(II)	140	[25]
Ti_3_C_2_T_*x*_	Cr(VI)	250	[26]
TiO_2_–C	Cr(VI)	225	[27]
Ti_3_C_2_	Ba(II)	9.3	[28]
DL- Ti_3_C_2_T_*x*_	Cu(II)	78.45	[29]
Magnetic Ti_3_C_2_T_*x*_	Hg(II)	1128.41	[30]
V_2_CT_*x*_	U(VI)	174	[35]
hydrated Ti_3_C_2_T_*x*_	U(VI)	214	[36]
Ti_2_CT_*x*_	U(VI)	470	[37]
Ti_2_CT_*x*_/PDDA	Re(VII)	363	[38]
Ti_2_CT_*x*_-hydrated	Th(IV)	213.2	[39]
HTNs	Eu(III)	200	[40]
Fe_3_O_4_/MXene	PO_4_^−3^	9.42	[31]
Ti_3_C_2_T_*x*_	BrO_3_^−^	321.8	[32]

*PEI/PAA* polyethylene polyimide/poly (acrylic acid), *MB* methylene blue, *ST* safranine T, *NR* neutral red, *MO* methylene orange, *RhB* rhodamine B, *DL-MXene* delaminated MXene, *PDDA* poly (diallyl dimethylammonium chloride), *HTNs* hierarchical titanate nanostructures

### Chemical adsorbent of radioactive ions

The radioactive ions are produced after the radioactive nuclides are consumed at the nuclear power plant or other nuclear-related technologies such as mining or medical research [33]. As the leakage of the radioactive ions to the surrounding soil and groundwater becomes a serious hazard, the efficient nuclear waste treatment and environmental management are necessary. Here, MXenes, based on their surface redox-ability and tunable adsorption properties, have proven to be relatively advantageous for adsorbing radioactive heavy metal ions [34]. Wang et al. [35] demonstrated the capability of multilayered V_2_CT_*x*_ MXene towards efficient adsorption of uranium ions U(VI) from aqueous solutions. The V_2_CT_*x*_ exhibited an outstanding adsorption capability with U(VI) uptake capacity of 174 mg g^–1^, fast sorption kinetics, and desirable selectivity. The DFT calculation suggested that the uranyl ions prefer to coordinate with the –OH groups bonded to the V-sites in the MXene nanosheets via bidentate inner-sphere complexes, reflecting the importance of the surface functionalization. In another attempt, Wang et al. [36] synthesized a series of hydrated and dry Ti_3_C_2_T_*x*_ for the fast removal of U(VI) from aqueous solutions. In comparison to the dry counterpart, the hydrated MXene showed the efficient removal of U(VI) with an adsorption capacity of 214 mg g^−1^, mainly due to its more flexible nature and much larger interlayer spacing. This study identified Ti_3_C_2_T_*x*_ as a promising candidate for U(VI) capture and encapsulation. In similar content, Wang et al. [37] showed the capability of 2D Ti_2_CT_*x*_ MXene to remove uranium via a sorption-reduction strategy. The batch experiments demonstrated that the Ti_2_CT_*x*_ could exhibit excellent U(VI) removal over a wide pH range, with an uptake capacity of 470 mg g^–1^ at pH 3.0. The study suggested that the Ti_2_CT_*x*_ material could also be a potential candidate to develop permeable reactive barriers with potential applications in treating wastewaters affected by uranium mining.

Other than uranium, MXenes have also shown promising adsorption capability towards heavy-metal ions in nuclear waste such as Re (IV), Th (IV), and Eu (III). The MXene adsorption efficiency towards Re (IV) was evaluated by Wang et al. [38]. The study utilized a three-dimensional (3D) MXene-polyelectrolyte nanocomposite for the enhanced removal of perrhenate ion (ReO_4_^−^). The introduction of poly (diallyl dimethylammonium chloride) (PDDA) into Ti_2_CT_*x*_-MXene regulated the surface charge and improved the stability. The Ti_2_CT_*x*_/PDDA composite achieved a removal capacity of up to 363 mg g^–1^ for Re (VII) with fast sorption kinetics and good selectivity in the presence of competing anions such as Cl^–^ and SO_4_^2–^ with a concentration 1800 times higher than Re (VII). Li et al. [39] investigated the performance of 2D Ti_2_CT_*x*_ MXene for Th(IV) removal and demonstrated that the hydrated MXene had a superior adsorption capability compared to the dry-counterpart. In this case, the sorption equilibrium was achieved within 720 min, with a maximum sorption capacity of 213.2 mg g^−1^. The hydrated Ti_2_CT_*x*_ exhibited an excellent selectivity over a range of competing ions, and the sorption process was highly pH-dependent with no influence from the overall ionic strength. The thorium (Th (IV)) sorption mechanism was determined to be an inner-sphere complexation originating from the strong binding affinity of Ti–OH towards Th (IV). In another study, hierarchical titanate nanostructures (HTNs) were derived from in situ chemical conversion of 2D Ti_3_C_2_T_*x*_-MXene crystal [40]. The formed HTNs were highly stable with a sorption capacity of 200 mg g^−1^ for Eu (III), owing to the well-maintained layered structure and abundant exchangeable guest cations. The sequestration of Eu (III) was realized by forming inner-sphere surface complexes in the nano-confined space, as evidenced by the decrease of Eu–O distances and coordination numbers. The finding of the inner-sphere complexation induced by Ti − O/Ti − OH coordination and confinement effect provided new insights into the interaction mechanism between radionuclides and titanates.

### MXenes in membrane separation of pollutants

Membrane separation technology is a practical route for desalination and wastewater treatment. The ideal desalination and water treatment membranes should have high flux, high selectivity, stability, and resistance against fouling and chlorine-like chemicals [41]. The membrane should also be thin enough with sufficient mechanical stability to maximize water permeability with a constant salt rejection rate. At present, 2D carbon nanomaterials, such as graphene and graphene oxide (GO), are promising materials with molecular and ionic sieving capability. Although suitable for many applications, the flux of GO membranes is relatively small due to the reduced interlayer spacing under pressure. Here, MXenes, with the tunable surface chemistry and manageable interlayer spacing, have been proven to be highly practical.

The MXene-based water treatment membrane was firstly reported by Ren et al. [42]. The membrane was produced using a vacuum-assisted filtration method, where 2D Ti_3_C_2_T_*x*_ MXene nanosheets were assembled into a freestanding membrane. Unlike graphene or GO, the high hydrophilicity of Ti_3_C_2_T_*x*_ allows the presence of interlayer H_2_O, which promotes ultrafast water flux. The produced membrane was nonpermeable to cations with hydration radii larger than the MXene interlayer spacing (∼6 Å), and had good selectivity toward positively charged metal cations (like Li^+^, Na^+^, K^+^, Mg^2+^, Ca^2+^, Ni^2+^, and Al^3+^), and methylthioninium^+^ (MB^+^) dye cations. The proposed micrometer-thick membrane could reach 37.4 L m^−2^ h^−1^ bar^−1^ water flux with different sieving capabilities based on the target ions' hydration radius and charge.

**Fig. 4:**
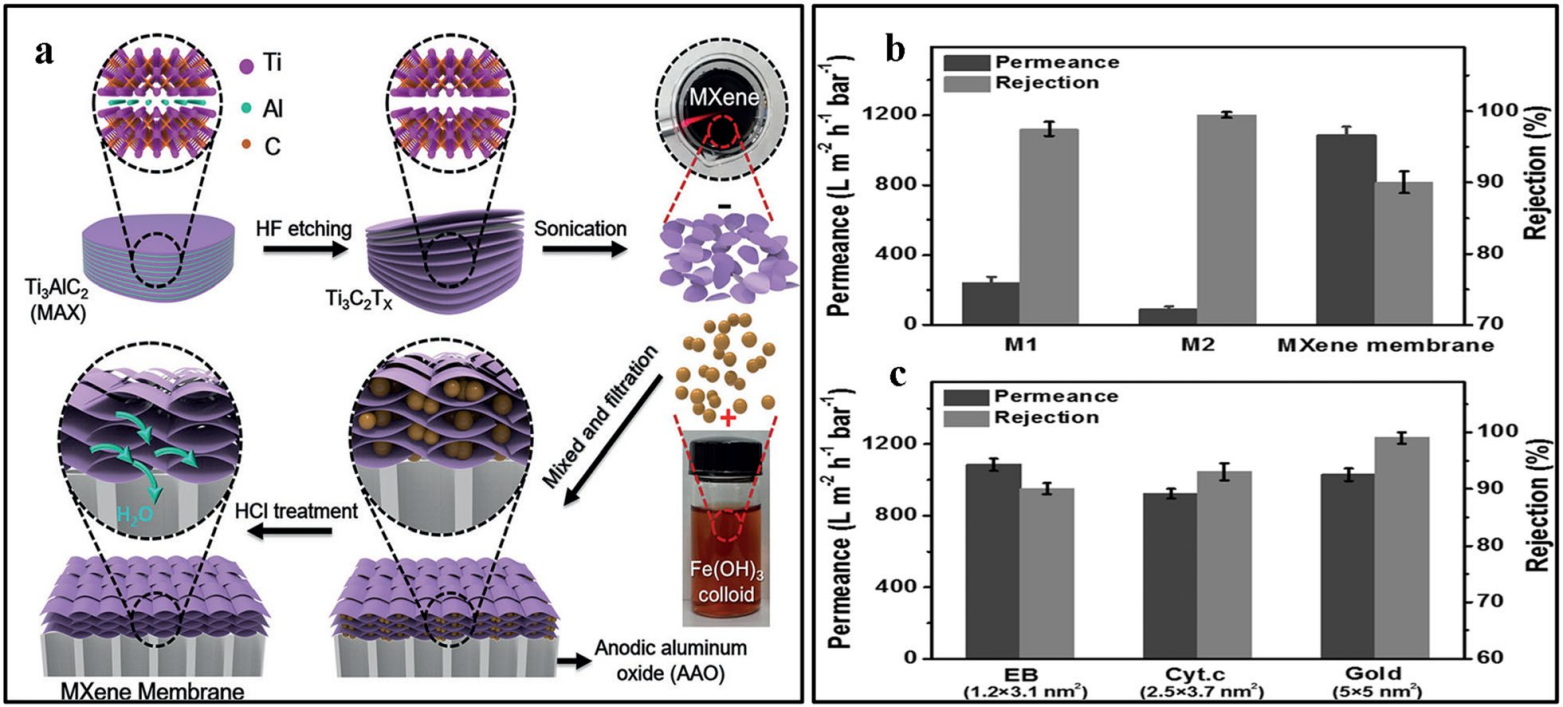
**a** Schematic diagram depicting the assembly of porous MXene membrane with Fe (OH)_3_ nanoparticles as template. **b** analytical comparison of M1, M2, and MXene membranes projected for EB molecules; **c** separation efficiency of MXene membranes estimated for various molecules of different sizes. Reprint with permission [43]. Copyright 2017,Wiley–VCH Verlag GmbH & Co. KGaA, Weinheim

Ding et al. [43] reported the use of porous MXene membrane, prepared using vacuum filtration with Fe(OH)_3_ nanoparticles as template (Fig. 4a). The obtained MXene membrane supported on anodic aluminum oxide substrate showed excellent water permeance of more than 1000 L m^−2^ h^−1^ bar^−1^ with a rejection rate of over 90% for molecules with sizes larger than 2.5 nm (Fig. 4b, c). The performance of the MXene-based membrane was superior to other membranes with a similar rejection rate.

GO-based membranes have proven their potential in gas and liquid separations. Improved performance could be achieved by coupling GO with MXene nanosheets. Kang et al. [44] attempted to integrate GO and MXene to prepare a 90 nm thick Ti_3_C_2_T_*x*_–GO composite membrane. After swelling, the prepared membrane’s lattice period was 14.28 Å, which corresponds to the interlayer spacing of around 5 Å, capable of allowing two layers of water molecules. The composite membrane effectively rejected the dye molecules with hydrated radii above 5 Å and the positively charged dye molecules during pressure-driven filtration at 5 bar. The layer configuration enabled the assembled membrane to achieve an excellent rejection rate of 68% for methyl red, 99.5% for MB, 93.5% for rose Bengal, and 100% for brilliant blue (hydrated radii of 4.87, 5.04, 5.88, and 7.98 Å, respectively). This study paved a new pathway to produce efficient separation membranes based on composite interlayer engineering. In this context, Han et al.[45] prepared an MXene-coated polyether sulfone (PES) ultrafiltration membrane with 0.2 MPa for dye desalination and wastewater treatment. The configured composite membrane had rough and dense surface layers and uniform element distribution. Interestingly, the rejection rates of dyes and inorganic salts relied on the integrated content of MXene. At optimum conditions, high rejection rates to Congo red dye (CR) (92.3%) and gentian violet (80.3%) were obtained, respectively, with a high flux of 115 L m^−2^ h^−1^ at 0.1 MPa. However, the rejection rate of the inorganic salts was lower than 23% with flux above 432 L m^−2^ h^−1^ at 0.1 MPa due to the loose lamellar structure of the composite membranes.

In another attempt to further improve the antifouling properties and increase the water flux of the MXene membrane, Pandey et al. [46] reported Ag nanoparticles-modified 2D Ti_3_C_2_T_*x*_ MXene (Ag@MXene) as an ultrafast water purification membrane. The Ag@MXene composite membranes with variable Ag loadings (0 ~ 35%) were prepared using self-reduction of silver nitrate on MXene sheets. The optimum performance of the Ag@MXene membrane was achieved when the thickness and average pore size was about 470 and 2.1 nm, respectively. The membrane exhibited an outstanding water flux of ∼420 L m^−2^ h^−1^ bar^−1^, which was relatively higher compared to its pristine counterpart. Also, the Ag@MXene membrane achieved favourable rejection to organic foulants like bovine serum albumin (100%), methyl green (92.3%) and RhB (79.9%), indicating its suitability for practical application.

MXene-based membranes have also been explored for air filtration. Gao et al. [47] reported a 2D MXene nanosheet-modified PAN fiber membrane for air purification. The introduction of Ti_3_C_2_ MXene nanosheets in PAN fiber enhanced the PM2.5 removal efficiency to ∼99.7% with a low-pressure drop of ∼42 Pa, together with the antibacterial activity. The high PM 2.5 removal efficiency can be ascribed to the large surface and accessible terminations of the MXene nanosheets, allowing fast and abundant adsorption of PM2.5 particles based on the strong interactive forces.

Table 2 summarizes the water purification performances of the MXene-based membranes. Although there are promising outcomes, specific challenges are still needed to overcome before MXenes could reach their full-potential in water and air filtration. The most crucial concern is the swelling of MXene, similar to any other 2D material, which is a limiting factor for the filtration of multiple-ions with diverse sizes at the same time. Moreover, this further influences the stability and recyclability of MXene-based films. Interlayer engineering is an efficient approach to overcome this issue where the adjustment of channel size by controlling the d-spacing of MXenes can allow the control of swelling and avoid structural collapse. This could promote the development of MXene-based membranes with high selectivity, extreme cyclability, and good capability to filter-out a wide range of pollutants.

**Table 2:** Performance of MXene-based membranes in water purification

Membranes	Pollutants	Experimental condition	Support layer	Thickness (nm)	Pure water flux L/(Bar·h·m^2^)	Removal (%)	Reference
Ti_3_C_2_T_*x*_	Li^+^ Na^+^ K^+^ Ni^2+^ Mg^2+^ Ca^2+^ AL^3+^ MB^+^	–	PVDF	1500	37.4	–	[42]
Ti_3_C_2_T_*x*_	Rhodamine B Evans blue Cytochrome [Fe(CN)_6_]^−3^ TMPyP BSA Au NPs	Dead-end/cross-flow C_o_ = 10–20 mg/L	AAO	200–1000	806 1084 1056 1120 921 790 1028	85 90 97 32 93 100 99	[43]
Ti_3_C_2_T_*x*_—GO	Brilliant blue Rose Bengal Methylene blue Methylene red MgSO_4_ NaCl	Dead-end C_o_ = 10 mg/L C_o _= 0.1 M	PC, nylon	20–90	0.23 0.67 0.3 2.1 2.35 2.25	100 93.5 99.5 61 < 11 < 11	[44]
Ti_3_C_2_T_*x*_	Congo red Gentian violet MgCl_2_ Na_2_SO_4_ NaCl	Dead-end C_o_ = 100–1,000 mg/L	PES	-	115 L m^−2^ h^−1^ 117.6 L m^−2^ h^−1^ 460 L m^−2^ h^−1^ 632 L m^−2^ h^−1^ 435 L m^−2^ h^−1^	92.3 80.3 23 13.2 13.8	[45]
Ag/Ti_3_C_2_T_*x*_	Rhodamine B Methyl green Bovine serum albumin NaCl MgCl_2_ AlCl_3_	dead-end C_o_ = 50–100 mg/L C_o_ = 2000 mg/L	PVDF	470	387.05 354.29 345.81 – – –	79.9 92.3 100 25.8 41.3 49.5	[46]
Ti_3_C_2_T_*x*_	PM2.5	–	PAN	1.25	–	99.7%	[47]

*PVDF* polyvinylidene difluoride, *AAO* anodic aluminum oxide, *PC* polycarbonate, *PES*: polyethersulfone, *PAN* polyacrylonitrile

### MXenes in photocatalytic degradation of pollutants

Photocatalytic degradation of organic pollutants is a promising environmental remediation technology. To date, several photoactive semiconductors, such as TiO_2_ [48], g-C_3_N_4_ [49], and CdS [50], have shown promising activity towards photodegradation of various organic pollutants. Although efficient, the photoexcited charge carrier’s fast recombination in these single semiconductor photocatalysts limits their practical application. Here, MXenes can serve as unique substrates to assemble advanced co-catalysts to effectively alleviate the charge-carrier recombination while enhancing the photocatalysts dispersibility and adsorption capability.

**Fig. 5:**
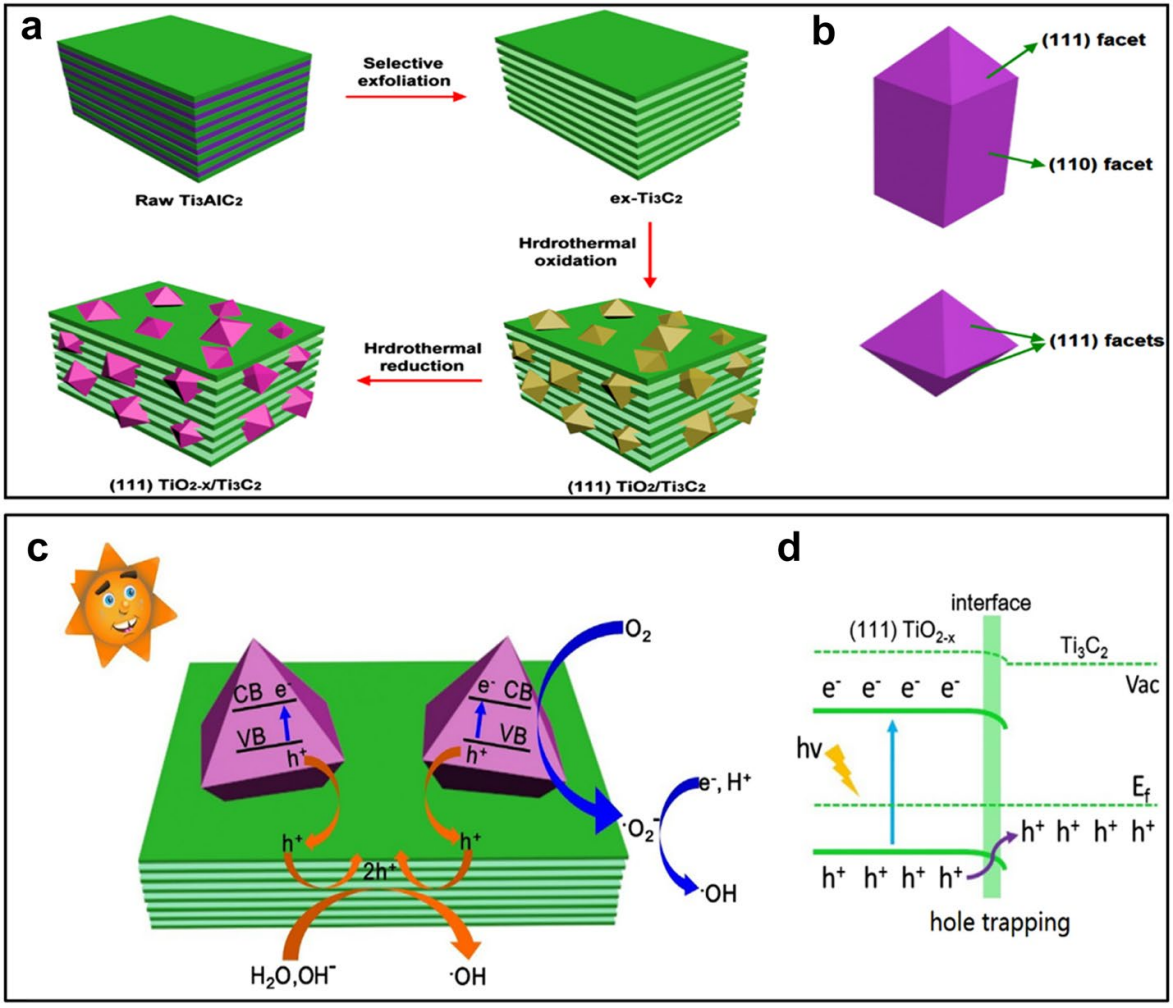
**a** Schematic of the formation of (111) TiO_2-x_/Ti_3_C_2_ nanocomposite with **b** the crystallographic orientations of rod-like and octahedral rutile. **c** Photogenerated charge carrier transfer mechanism and **d** the corresponding band alignments. Reprint with permission [53]. Copyright 2017, Elsevier Ltd

Mashtalir et al. [19] reported the first use of MXenes for the degradation of MB and AB80 dyes under ultraviolet (UV) light. In the absence of light, MXenes could adsorb 18% of MB and no AB80 up to 20 h, while under 5 h of irradiation, 62, and 81% degradation was achieved, respectively. The photo-activity, in this case, was the direct consequence of TiO_2_ produced after partial surface oxidation of the Ti_3_C_2_T_*x*_ MXene. The TiO_2_ provided the photocatalytic activity, whereas the under layered Ti_3_C_2_ served as an adsorbent bed, contributing to fast charge transport. Subsequently, the TiO_2_/Ti_3_C_2_ composites have been widely studied for photocatalytic application. Gao et al. [51] fabricated a TiO_2_/Ti_3_C_2_ composite for the photocatalytic degradation of MO dye. The composite exhibited an excellent photodegradation capability, where 98% of MO was completely mineralized in 30 min. Also, Peng et al. [52] prepared an MXene/TiO_2_ composite, in which TiO_2_ (001) facets were obtained via partial oxidation of Ti_3_C_2_ assisted by NaBF_4_. Here, Ti_3_C_2_ acted as a precursor to TiO_2_ and an active component to construct Schottky-junction with the (001) surface of the n-type semiconductor (TiO_2_). Under illumination, the heterojunction realized an ultra-low work function with spatial separation of photogenerated charge-carrier, achieving a photocatalytic degradation rate of 18.8 min^−1^ g^−1^ for MO dye. The same group [53] later reported a (111) TiO_2-x_/Ti_3_C_2_ nanocomposite, in which Ti^3+^-doped rutile TiO_2_ octahedrons with exposed active (111) facets were derived from 2D Ti_3_C_2_ sheets using hydrothermal oxidation followed by reduction via hydrazine hydrate (Fig. 5a, b). The 2D Ti_3_C_2_ served as a pathway for transferring photogenerated holes to promote the charge separation efficiency (Fig. 5c, d), whereas the exposed TiO_2_ (111) maximized the photocatalytic ability of the TiO_2_/Ti_3_C_2_ composite. The hybrid achieved enhanced MB dye degradation under visible light (75% of MB in 150 min). The TiO_2_/MXene composite has also been utilized for the photocatalytic degradation of pharmaceutical pollutant known as carbamazepine (CBZ) [54], which achieved a 98.67% degradation efficiency in 4 h. The kinetics of the photocatalytic reaction was determined to follow Langmuir–Hinshelwood kinetic model with a Kapp value of 0.0304 min^−1^ at pH 3.0.

The charge carrier recombination is a major limitation in semiconductor-based photocatalyst, restricting their long-term use in the photocatalytic degradation process. Here, the construction of heterojunction of semiconductor photocatalyst with other co-catalyst in a cascade-type configuration can readily minimize this charge-carrier recombination while improving the production efficiency of free radicals (˙O_2_^−^ and ˙OH), that are responsible for the mineralization of the target pollutant. In regard to CuO based photocatalyst, Lu et al.[55] proposed coupling of CuO with Ti_3_C_2_/TiO_2_ system to construct an efficient Ti_3_C_2_/TiO_2_/CuO heterojunction. The composite was prepared by decomposing a mixture of Ti_3_C_2_ MXene and cupric nitrate under an argon atmosphere, which later realized 99% degradation efficiency for MO dye in 80 min. Similarly, Wojciechowski et al. [56] studied the photocatalytic degradation of salicylic acid (SA) using the composites that were prepared using a combination of Ti_2_C MXene and metal oxide (such as TiO_2_, Ag_2_O, and PdO) and metal (like Ag, Pd, and Au) nanoparticles. Unexpectedly, the modification of MXene with noble metal nanoparticles did not bring any significant improvement besides serving as metal centers to accept electrons from the conductive band of TiO_2_ particles that were previously embedded in MXenes to produce Ti_2_C/TiO_2_/Ag_2_O composites. The highest SA degradation (95.8%) was achieved after 180 min of irradiation in the configuration of Ti_2_C/3%TiO_2_/1%Ag_2_O.

The complete conversion of Ti-based MXenes into TiO_2_@C composites has also been reported for the photocatalytic activity. Li et al. [57] fabricated TiO_2_@C nanosheets by high-energy ball milling of 2D Ti_2_CT_*x*_. In this case, Ti_2_CT_*x*_ served as a titanium and carbon source, and the formed TiO_2_ nanoparticles were uniformly distributed on the single- or few-layered carbon sheets. Unlike the pristine TiO_2_ and photocatalytic standard (P25), the TiO_2_@C nanosheets exhibited enhanced photocatalytic degradation of MB dye with a degradation efficiency of 85.7% in 360 min.

Metal sulfides are another major group of abundant and cheap minerals that have been coupled with MXenes to curb the charge-carrier recombination and achieve the high photocatalytic activity. Wang et al. [58] constructed a quasi-core–shell In_2_S_3_/TiO_2_@Ti_3_C_2_T_*x*_ heterostructure hybrid. The hybrid with a Ti_3_C_2_T_*x*_ content of 16 mg achieved an improved visible-light photocatalytic activity towards MO dye (92.1% MO in 60 min) with a degradation rate of 0.04977 min^−1^, which was 3.2 and 6.2 fold higher compared to pure In_2_S_3_ and pristine Ti_3_C_2_T_*x*_, respectively. Also, Liu et al. [59] developed a 2D CdS@Ti_3_C_2_@TiO_2_ nanohybrid using a facile hydrothermal process, which exhibited significantly enhanced visible-light-driven (λ ≥ 420 nm) degradation of sulfachlorpyridazine (SCP), and several dye contaminants (100% for RhB, MB, SCP, and phenol in 60, 88, 88, and 150 min, respectively).

Besides TiO_2_ derived from Ti-based MXenes, other photocatalysts have also been used to combine with MXenes to fabricate advanced co-catalyst materials. CeO_2_ is a widely studied photocatalyst with limited solar spectrum utilization. In this context, Zhou et al. [60] synthesized a CeO_2_/Ti_3_C_2_ nanocomposite by coupling well-dispersed CeO_2_ nanorods with Ti_3_C_2_ sheets under a hydrothermal process. Compared to its pristine counterparts, the composite exhibited an enhanced photocatalytic activity for the photodegradation of RhB under UV-light irradiation, where 75% of RhB was degraded within 90 min.

Hematite (α-Fe_2_O_3_) is an attractive photocatalyst due to its accessibility, magnetic properties, corrosion resistance, low cost, and visible light absorption ability. Zhang et al. [61] prepared an α-Fe_2_O_3_/Ti_3_C_2_ MXene composite using an ultrasonic-assisted self-assembly approach. In this case, 2D α-Fe_2_O_3_ nanosheets were anchored to the Ti_3_C_2_ MXene, constructing a heterostructure with high visible absorption capability and ultrahigh degradation efficiency of 98% in 120 min for MB dye.

The ferrite-based materials are a promising family of photocatalyst, but their inherent narrow-bandgap limits their light absorption and photocatalytic ability. In this regard, Tariq et al. [62] doped Gd^3+^ and Sn^4+^ within BiFeO_3_ (Bi_*1–x*_Gd_*x*_Fe_*1–y*_ Sn_*y*_; BGFSO), and then combined with 2D Ti_3_C_2_T_*x*_-MXene to improve the overall surface area and construct a charge-transfer network. The BGFO-20Sn/MXene nanohybrid demonstrated 100% photocatalytic degradation ability towards CR dye in 120 min with minimum charge-carrier recombination. Similarly, Iqbal et al. [63] hybridized a La, Mn-codoped BiFeO_3_ with Ti_3_C_2_T_*x*_ using a sol–gel method. The constructed composite delivered 92% degradation of CR dye just within 10 min under visible light irradiation. The spinel ferrites (such as CuFe_2_O_4_) are another type of photocatalyst known for their high plasticity and excellent chemical stability. However, the fast aggregation of the spinel ferrites based on their strong magnetic nature results in a diminished active site ratio. To overcome this issue, Cao et al. [64] anchored CuFe_2_O_4_ nanoparticles onto Ti_3_C_2_ MXene nanosheets using an in situ sol-hydrothermal method. The CuFe_2_O_4_/MXene photocatalyst was used for the photocatalytic degradation of pharmaceutical pollutants such as sulfamethazine (SMZ) under visible light, which achieved a degradation rate of 59.4%, with increased charge-carrier lifetime and photostability.

MXenes have also been used as promising support materials to increase light harvesting characteristics of the tungsten-based photocatalysts. Fang et al. [65] proposed an Ag_2_WO_4_/Ti_3_C_2_ composite, where the integration of the conductive Ti_3_C_2_ allowed the heterogeneous distribution of the Ag_2_WO_4_ catalyst. The Ti_3_C_2_ promptly enhanced the catalytic activity and raised the corrosion resistance ability of Ag_2_WO_4_, besides acting as a conductive substrate to improve the photoinduced charge transportation charge-carrier lifetime (34.5 μs). The Ag_2_WO_4_/Ti_3_C_2_ composite exhibited robust photocatalytic activity toward tetracycline hydrochloride (TC) and sulfadimidine (SFE) with 62.9% and 88.6% degradation efficiency, respectively. Also, Cuia et al. [66] synthesized a 2D/2D Bi_2_WO_6_/Nb_2_CT_*x*_ hybrid photocatalyst using a simple hydrothermal process, which exhibited promising photocatalytic activity against RhB, MB, and tetracycline hydrochloride (TC-HCl). In this case, the 2D/2D interfacial configuration led to the improved separation of photogenerated carriers and degradation efficiency of 99.8, 92.7, 83.1% for RhB, MB, TC-HCl, respectively. In another case, Cai et al. [67] fabricated an Ag_3_PO_4_/Ti_3_C_2_ Schottky catalyst using a self-assembly strategy. The composite achieved a high photocatalytic rate for the degradation of 2,4-dinitrophenol, which was 2.5-fold higher than the Ag_3_PO_4_/graphene and 10 times higher than the pristine Ag_3_PO_4_. The superior photocatalytic activity, in this case, was attributed to the intimate contact between the hybrid components, and the constructed Schottky barrier was responsible for limiting the charge-carrier recombination.

Among the widely used photocatalyst, graphitic carbon nitride (g-C_3_N_4_) is a well-known metal-free catalyst with high stability and adequate bandgap. However, g-C_3_N_4_ suffers from a short charge-carrier lifetime, which leads to the gradual diminishing photocatalytic activity. In this regard, Liu et al. [68] developed a visible-light photocatalyst composed of g-C_3_N_4_ and Ti_3_C_2_ using an evaporation-induced self-assembly method. The g-C_3_N_4_/Ti_3_C_2_ composite exhibited an excellent photocatalytic activity towards pharmaceutical pollutant know as ciprofloxacin (CIP) with 100% degradation within 150 min. The degradation profile followed a pseudo-first-order kinetic, where the CIP decomposition was 2.2 folds faster than the pristine g-C_3_N_4_ under visible-light irradiation. To further improve the performance of g-C_3_N_4_/Ti_3_C_2_ composite, Ding et al. [69] suggested the integration of plasmonic particles, e.g. Ag. In this case, the layered Ti_3_C_2_ MXene acted as a supporter and reductant to produce Ag nanoparticles, which later contributed to the photogenerated electrons transportation. The constructed Schottky junction in synergism with Ag nanoparticles improved the optical absorption and electron-donating ability, making the Ag-integrated g-C_3_N_4_/Ti_3_C_2_ composite realize 81.8% degradation ability for aniline. The photodegradation performance was 4 and 8 times higher than the pristine g-C_3_N_4_ and Ti_3_C_2_, respectively.

**Fig. 6:**
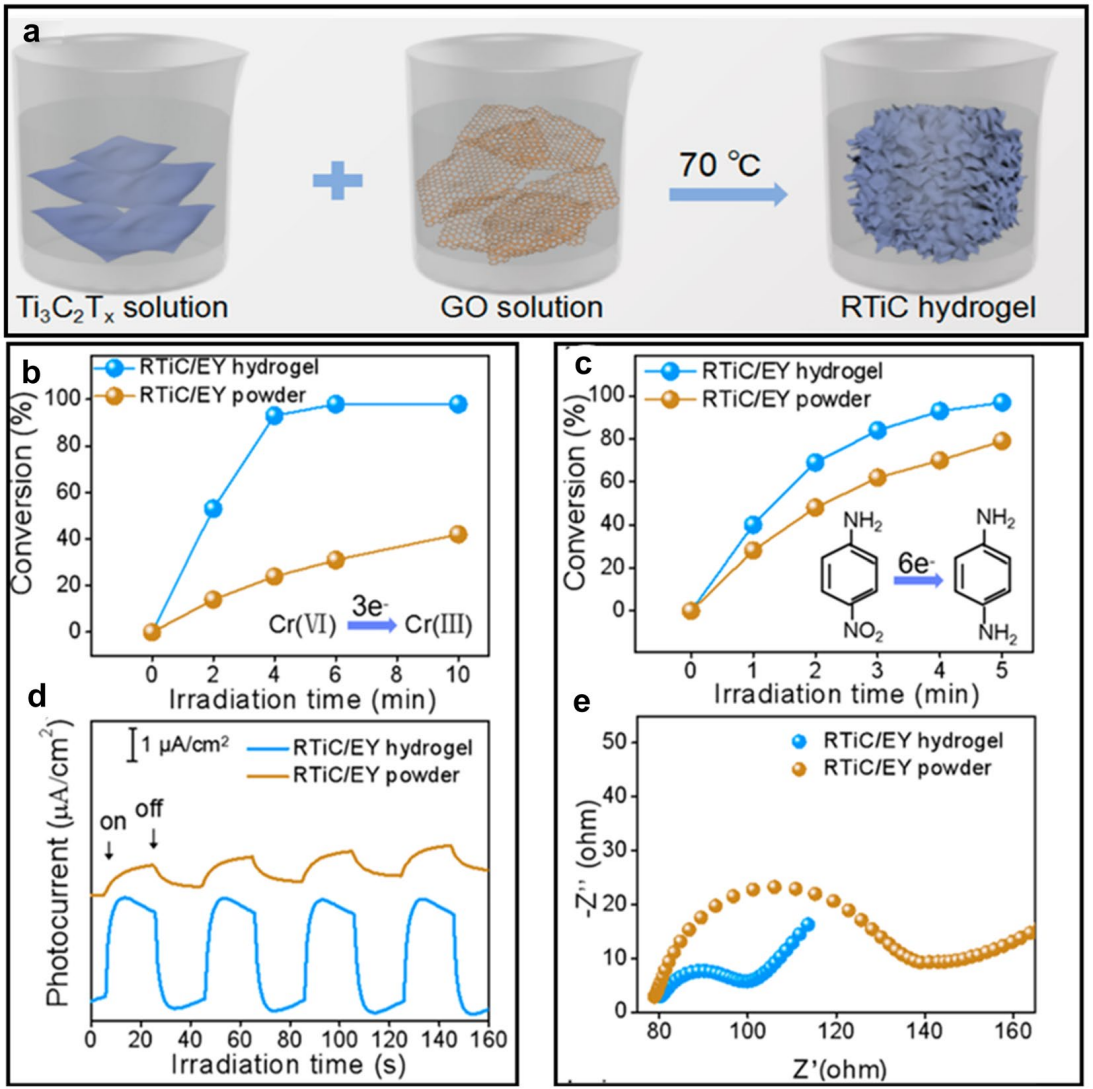
**a** Fabrication process of the RTiC with photocatalytic reduction of **b** Cr(VI) and **c** 4-NA using RTiC/EY hydrogel and the powdered counterpart with **d** corresponding transient photocurrent spectra and **e** Nyquist plots from the electrochemical impedance spectroscopy. Reprint with permission [73]: copyright, 2018, American Chemical Society

The transformation of 2D layered MXenes into 3D macroscopic hydrogel has recently shown tremendous photocatalysis potential [70, 71]. When MXenes are assembled into hydrogel systems, they offer exciting and versatile platforms to design MXene-based soft materials with tunable properties [72]. In this regard, Chen et al. [73] reported an organics-free and straightforward strategy to construct a 3D Ti_3_C_2_T_*x*_-based hydrogel (RTiC) using a GO-assisted self-convergence process, as shown in Fig. 6a. The hydrogel was then integrated with Eosin Y (photosensitizer) thereby allowing the composite gel to demonstrate enhanced photoactivity towards Cr(VI) and 4-Nitrophenol (4-NA), with 99.3 and 97% degradation efficiencies in 10 and 5 min, respectively (Fig. 6a–d). In addition, bismuth oxyhalides have also been coupled with MXene for improved photocatalytic performance. Cui et al. [74] used Ti_3_C_2_T_*x*_ MXene material to construct a BiOBr_0.5_I_0.5_/MXene heterostructure photocatalyst, which realized 100% and 50% degradation for RhB and phenol, respectively. Table 3 summarizes all the MXene-based photocatalysts reported for the photodegradation of the environmental pollutants.

**Table 3:** Photodegradation performance for the organic dyes and some chemical compounds using MXene and MXene-based composite photocatalysts

MXene-based photocatalyst	Pollutant	Light source	Concentration and volume	Degradation efficiency	Reference
Ti_3_C_2_T_*x*_	MB AB80	UV-C lamp (254 nm)	0.012 mg L^−1^, 40 mL 0.06 mg L^−1^, 40 mL	81% in 5 h 62% in 5 h	[19]
TiO_2_/Ti_3_C_2_	MO	175 W Hg lamp	20 mg L^−1^, 100 mL	98% in 30 min	[51]
(001) TiO_2_/Ti_3_C_2_	MO	300 W Hg lamp	20 mg L^−1^, 200 mL	97.4% in 50 min	[52]
(111) TiO_2_/Ti_3_C_2_	MB MO	500 W Xe lamp	20 mg L^−1^, 200 mL	75% in 150 min	[53]
TiO_2_/Ti_3_C_2_T_*x*_	CBZ	100 W medium pressure lamp	5 mg L^−1^, 10 mL	98.67% in 4 h	[54]
Ti_3_C_2_/TiO_2_/CuO	MO	175 W Hg lamp	20 mg L^−1^, 100 mL	99% in 80 min	[55]
Ti_2_C/TiO_2_/Ag	SA	150 W Hg lamp	100 μM, 400 mL	97.1% in 3 h	[56]
TiO_2_@C	MB	500 W Hg lamp	20 mg L^−1^, 50 mL	85.7% in 360 min	[57]
In_2_S_3_/TiO_2_@ Ti_3_C_2_T_*x*_	MO	300 W Xe lamp	20 mg L^−1^, 100 mL	92.1% in 60 min	[58]
CdS@Ti_3_C_2_@TiO_2_	RhB MB SCP Phenol	Visible light with 300 mW cm^−2^	20 mg L^−1^, 200 mL	100% in 60 min 100% in 88 min 100% in 88 min 100% in 150 min	[59]
CeO_2_/Ti_3_C_2_	RhB	500 W Hg lamp	20 mg L^−1^, 50 mL	75% in 90 min	[60]
Fe_2_O_3_/Ti_3_C_2_	RhB	500 W Xe lamp	10 mg L^−1^, 100 mL	98% in 120 min	[61]
BGFO-20Sn/MXene	CR	300 W Xe lamp	**–**, 100 mL	100% in 2 h	[62]
BLFMO/MXene	CR	double beam UV/Vis–NIR spectrophotometer (Cary 5000, Varian)	–	~ 93% in 10 min	[63]
CuFe_2_O_4_/MXene	SMZ	300 W Xe lamp	40 mg L^−1^, –	59.4% in 60 min	[64]
Ag_2_WO_4_/Ti_3_C_2_	TC SFE	300 W Xe lamp	20 mg L^−1^, –	62.9% in 40 min 88.6% in 40 min	[65]
Bi_2_WO_6_/Nb_2_CT_*x*_	RhB, MB TC-HCl	500 W Xe lamp	15 mg L^−1^, 100 mL	99.8% in 90 min 92.7% in 90 min 83.1% in 120 min	[66]
Ag_3_PO_4_/ Ti_3_C_2_	MO 2,4-DNP TC-H TPL CPL	300 W Xe lamp	20 mg L^−1^, 50 mL	–	[67]
g-C_3_N_4_/Ti_3_C_2_	CIP	500 W Xe lamp	20 mg L^−1^, 50 mL	100% in 150 min	[68]
g- C_3_N_4_/ Ti_3_C_2_-AgNPs	Aniline	–	–	81.8% in 8 h	[69]
Ti_3_C_2_T_*x*_/GO/EY	Cr(VI) 4-NA	300 W Xe arc lamp	10 mg L^−1^, 20 mL	99.3% in 10 min 97% in 5 min	[73]
BiOBr_0.5_I_0.5_/ Ti_3_C_2_T_*x*_	RhB Phenol	300-W Xe lamp	20 mg L^−1^, 100 mL 10 mg L^−1^, 100 mL	100% in 40 min 50% in 5 h	[74]

*BGFO-20Sn* Gd and Sn co-doped bismuth ferrite BiFeO_3_ nanoparticles, *BLFMO* La and Mn co-doped bismuth ferrite BiFeO_3_ nanoparticles, *CR* Congo red, *MB* methyl blue, *AB80* acid blue 80, *MO* methyl orange, *CBZ* Carbamazepine, *RhB* Rhodamine B, *SCP* sulfachloropyridazine, *SA* salicylic acid, *2,4-DNP* 2,4-Dinitrophenol, *TC-H* tetracycline hydrochloride, *TPL* thiamphenicol, *CPL* chloramphenicol, *SMZ* sulfamethazine, *SFE* sulfadimidine, *CIP* ciprofloxacin, *4-NA* 4-nitroaniline

**Fig. 7:**
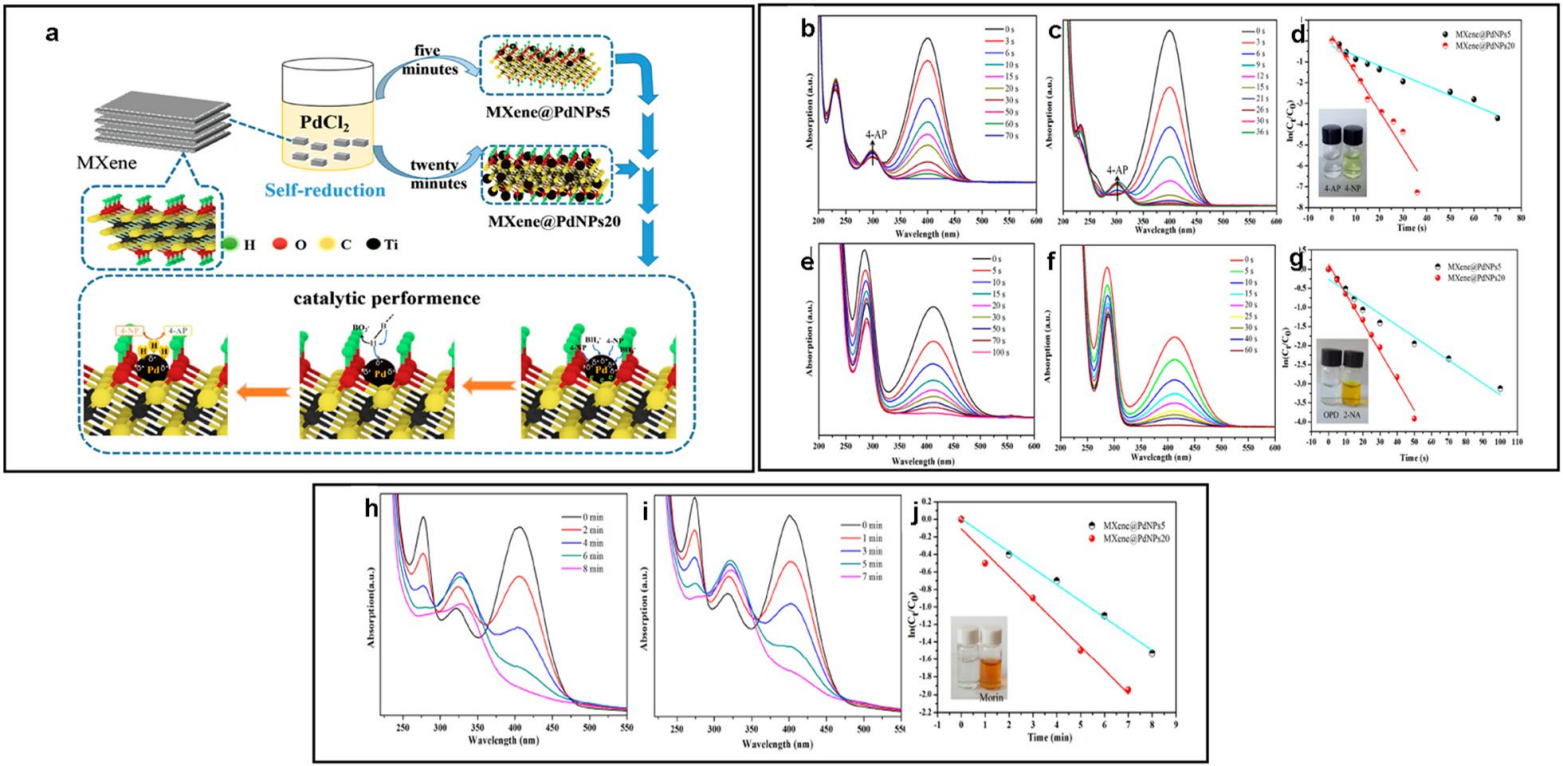
**a** Scheme showing the self-reduction formation of Pd nanoparticles on MXene sheets. UV-spectra and the corresponding rate kinetic plots obtained using the Pd/MXene composite catalysts of **b**–**d** 4-nitrophenol (4-NP), **e**–**g** and nitroaniline (2-NA) and **h**–**j** morin. Reprint with permission [79]. Copyright 2019 by the authors. Licensee MDPI, Basel, Switzerland

The use of MXenes has also been realized in the catalytic removal/degradation of environmental pollutants without irradiation. In this case, the catalytic system is configured to improve the oxidants overall performance or reductant to achieve complete degradation or mineralization of the pollutant. In this context, Li et al. [75] proposed MXene–COOH@(PEI/PAA)_n_@Au composite with hierarchical core–shell structure, which exhibited high catalytic activity towards nitro compounds, such as 2-nitrophenol (2-NP) and 4-nitrophenol (4-NP), in the presence of NaBH_4_ (reductant). In this case, the complete degradation of 2-NP and 4-NP was achieved in 36 and 30 min, respectively. Moreover, the catalyst was highly stable with maintained catalytic ability even after 8 continuous catalytic cycles. Xie et al. [76] prepared Pd/Ti_3_C_2_T_*x*_ graphene hydrogels with a 3D interconnected porous structure using a facile two-step process. The as-prepared Pd/Ti_3_C_2_T_*x*_ graphene hydrogels had a prominent porous structure, which could easily buffer the swelling of Ti_3_C_2_T_*x*_ during the catalytic process. Therefore, the mechanically robust Pd/Ti_3_C_2_T_*x*_ graphene hydrogels exhibited high activity, easy separability, and good cyclability. In another case, Lu et al. [77] synthesized a Co_3_O_4_/Ti_3_C_2_ MXene nanocomposite using a simple solvothermal method. The Co_3_O_4_/Ti_3_C_2_ nanocomposite exhibited an outstanding degradation efficiency for MB and RhB dyes (128.91 mg g^−1^ of MB in 300 min, 47.076 mg g^−1^ of RhB in 100 min) with maintained catalytic capability for 8 consecutive catalytic cycles. Besides, Liu et al. [78] prepared a sandwich-like Co_3_O_4_/Ti_3_C_2_T_*x*_ composite via a one-pot approach, which exhibited an outstanding catalytic degradation ability towards bisphenol A (BPA) in the presence of peroxymonosulfate (PMS, oxidant). Among the optimum content, the Co_3_O_4_/MXene-20% exhibited the highest removal capability, with 95% BPA degradation within 7 min. Additionally, Yin et al. [79] proposed a Pd/Ti_3_C_2_T_*x*_ composite for the reductive degradation of nitro compounds and morin. Figure 7a shows the scheme for the self-reductive formation of Pd nanoparticles on MXene, where the growth of the Pd particles was controlled by adjusting the reaction time. The Pd/MXene composite demonstrated excellent catalytic degradation ability against 4-nitrophenol (4-NP), and nitroaniline (2-NA) in the presence of NaBH_4_ with the reaction rate of 0.180 and 0.089 s^−1^, respectively (Fig. 7b–g). The catalyst maintained its robust activity even after 8 consecutive cycles, with a conversion rate of more than 94 and 91.8% for 4-NP and 2-NA, respectively. The Pd/MXene catalyst was capable of degrading mulberry pigment (morin) (Fig. 7h–j), but in this case, the degradation efficiency was relatively low compared to that achieved for 4-NP and 2-NA. Table 4 summarizes the MXene-based catalysts without irradiation along with their analytical parameters and efficiencies.

**Table 4:** Catalytic degradation performance of MXene and MXene-based composite catalysts for some pollutants

MXene-based catalyst	Pollutant	Concentration & volume	Oxidant/reductant	Catalytic efficiency	Reference
Ti_3_C_2_ –COOH@(PEI/PAA)@AuNPs	2-NA 4-NP	5 mM, 2 mL 5 mM, 2 mL	Fresh NaBH_4_ (20 mL, 0.01 M)	100% in 60 min 100% in 57 min	[75]
Ti_3_C_2_-Co_3_O_4_	MB RhB	12.5 mg/L,100 mL 5 mg/L,100 mL	H_2_O_2_ (30%, 15 mL)	128.91 mg g^−1^ in 240 min 47.076 mg g^−1^ in 80 min	[77]
Co_3_O_4_/MXene	Bisphenol A	20 mg/L,100 mL	Peroxymonosulfate (100 mL, 0.3 g/L)	95% in 7 min	[78]
Ti_3_C_2_@PdNPs20	2-NA 4-NP Morin	5 mM, 2 mL 5 mM, 2 mL 3 mM, 40 µL	Fresh NaBH_4_ (20 mL, 0.01 M) Fresh H_2_O_2_ (80 µL, 0.2 M)	100% in 60 s 100% in 36 s 100% in 7 min	[79]

*2-NA* nitroaniline, *4-NP* 4-nitrophenol

### MXenes in electrocatalytic sensors designed for pollutant detection

The detection of toxins/pollutants is the first step to environmental remediation. The electrocatalytic platform is an attractive technique for direct/indirect analysis of various contaminants based on its simplicity, university, and selectivity. The sensitivity of the electrocatalytic sensor directly relies on the catalytic component of the devised sensor. Thus, an electrocatalytic material with a robust active surface, sufficient conductivity, and high redox activity could significantly enhance the sensor's sensitivity and selectivity towards a particular toxin. Recently, MXenes have gathered substantial attention from the sensor's community based on their tunable surface chemistry, hydrophilicity, and active functionalization [80]. Additionally, MXene could comfortably be decorated/hybridized with other active components such as metal nanoparticles and carbon-based materials to achieve enhanced sensitivity and selectivity. In regard to toxic ions, Rasheed et al. [81] realized the application of 2D MXene sheets in redox-based catalytic detection of bromate ions. The spectroscopic analysis revealed that the adsorption of bromate ions over MXene sheets resulted in the simultaneous reduction of bromate and partial oxidation of MXenes. The constructed sensor exhibited robust sensitivity, with a detection limit of 41 nM for the working window of 50 nM-5 μM. The study provided evidence that MXene sheets could be directly used in redox-based sensor systems. In another case of detecting toxic metals, Cheng et al. [82] demonstrated the use of PANI-modified Ti_3_C_2_ slices for the electrochemical determination of mercury ions. The PANI-Ti_3_C_2_ composite with their co-coupling realized a linear detection range between 0.1 and 20 μgL^−1^ with a low detection limit of 0.017 μgL^−1^.

The poor electron transfer is an issue of acetylcholinesterase (AChE)-based electrocatalytic sensors for low-concentration organophosphate pesticides (OPs) detection. Jiang et al. [83] utilized MXene to construct a multi-component system, which could potentially serve as both an immobilization matrix for AChE and a conductive bed to facilitate charge transfer at the electrode interface. The devised AChE-based electrocatalytic biosensor relied on the enzymatic inhibition pathway, whereas the combination of Ti_3_C_2_T_*x*_ nanosheets with Ag nanoparticles increased the active interfacial sites. Thus, the hybrid system showed a good affinity towards acetylthiocholine chloride (ATCl) with an apparent Michaelis–Menten constant (Kmapp) value of 257.67 μM. The catalytic sensor could detect malathion in a linear range from 10^−14^ to 10^−8^ M, with satisfactory selectivity, acceptable reproducibility, and excellent stability. The devised sensor was also applicable for detecting malathion in a real sample (tap water) with satisfactory recoveries.

**Fig. 8:**
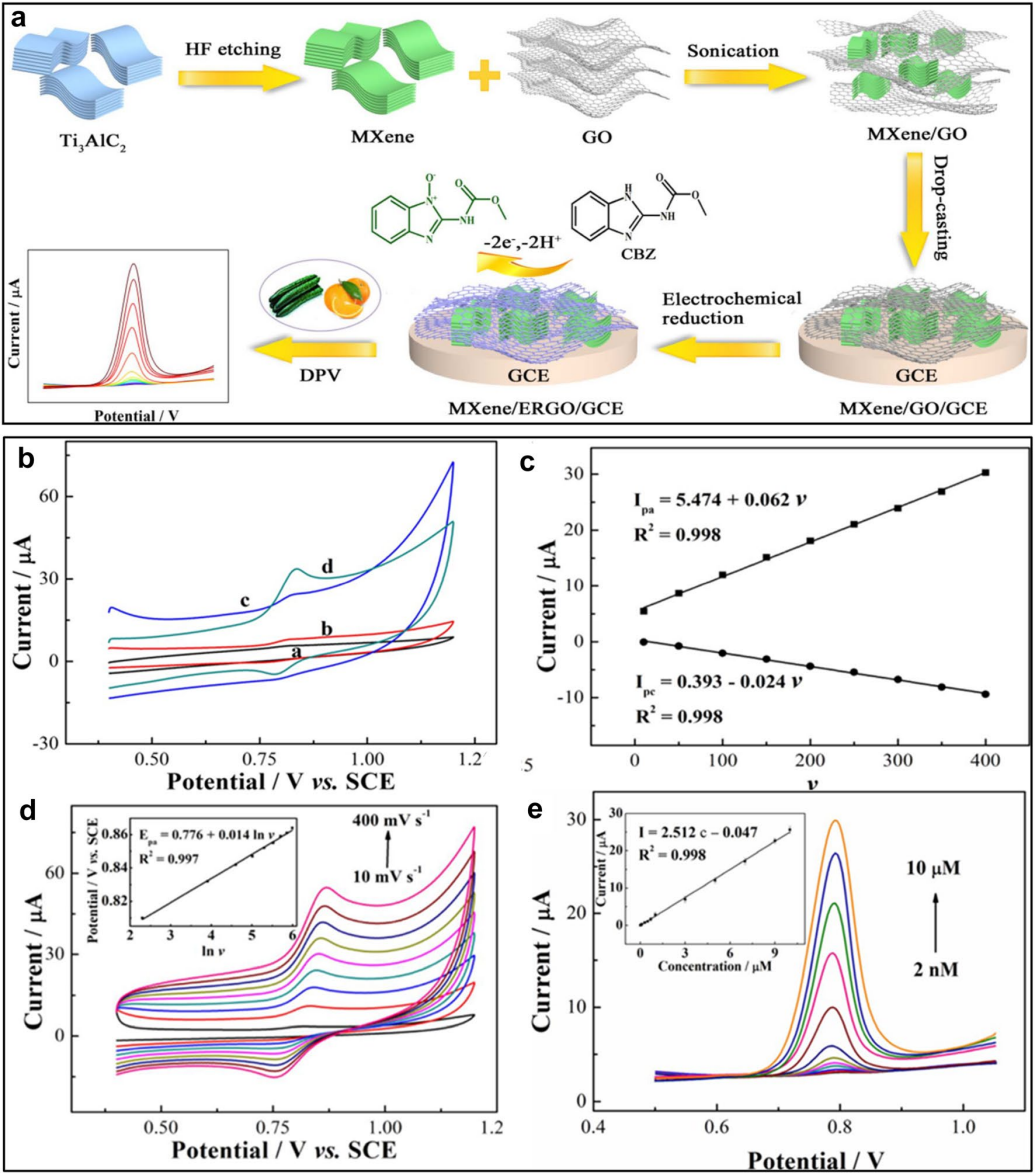
**a** Schematic illustration showing the preparation steps for MXene/rGO composite and its deposition on GCE for electrocatalytic sensing of CBZ drug. **b** CV profiles of the composite with CBZ (5.0 μM) in 0.1 M phosphate buffered Saline (PBS) (pH 7.0) in reference to bare GCE and other compositional competitors; **c** the corresponding relationship of peak current and natural log of scan rate **d** The variation of CV scans with different scan rate in the range of 10 to 400 mVs^−1^ and. **e** DPV plots for CBZ at concentrations of 0.002, 0.007, 0.05, 0.07, 0.1, 0.3, 0.5, 0.7, 1.0, 3.0, 5.0, 7.0, 9.0 and 10.0 μM, respectively, with inset showing the corresponding linear fit calibration. Reprint with permission [85]. Copyright 2019 The Electrochemical Society

In another study, Wu et al. [84] proposed the direct use of Ti_3_C_2_T_*x*_ nanosheets for the electrocatalytic detection of carbendazim (CBZ). The computational studies indicated the importance of -F termination of Ti_3_C_2_T_*x*_ in electrochemical sensing. The device could detect CBZ in a range of 50 nM to 100 µM with a low detection limit of 10.3 nM and high selectivity in the presence of fenamiphos (10 folds), ametryn (10 folds), and metal ions (50 folds). Xie et al. [85] also proposed an MXene/rGO composite for CBZ. The composite was prepared by coupling the MXene and GO in a solution system followed by the electrochemical reduction of GO to rGO (Fig. 8a). The conductive network of rGO packed with MXene layers constructed a platform with enhanced catalytic activity and improved signal production ability based on fast charge-transport pathways and increased active surface sites. Consequently, the MXene/rGO exhibited a sensitive signal response towards CBZ in a wide linear range of 2.0 nM-10.0 μM and a low detection limit of 0.67 nM (Fig. 8b–e). The configured electrocatalyst was also capable of working in the organic matrix, such as cucumber and orange juice samples.

The MXene-based composite configurations have also been explored for detecting environmental pollutants. The high conductivity, 2D structure, large surface area, hydrophilicity and mechanical flexibility make MXenes to be a potential substrate to assemble hybrid composites [86]. In addition, the tunable surface chemistry and controlled layered structure of MXene (single or multi-layer) further allow MXene-based composites to show desirable characteristics, in favor of enhancing the sensitivity and selectivity of the sensors [87]. In this context, Huang et al. [88] utilized the MXene/MWCNT composite for the simultaneous detection of catechol (CT) and hydroquinone (HQ). The overlapping oxidation peaks of CT and HQ are a challenge when it comes to their precise detection. Here, the MXene, coupled with MWCNT, enabled separated signals for both toxins with an ultra-low detection limit of 6.6 and 3.9 nM for HQ and CT, respectively. The high conductivity and redox ability of the MXene in synergism with MWCNT enabled the selective oxidation of the CT and HQ with a significant potential difference. The devised sensor successfully detected HQ and CT in industrial wastewater with a recovery rate of 96.9 ~ 104.7% and 93.1 ~ 109.9%, respectively. Similarly, Wu et al. [89] used chitosan-modified MXene platform for the immobilization of tyrosinase, which was used to detect phenol. The 2D laminar structure and favourable functionality of the MXene enabled the chitosan molecules to be anchored easily, facilitating the immobilization of tyrosinase enzyme. The detection mechanism relied on the tyrosinase capability to oxidize phenol to o-quinone, which was later electrochemically reduced to polyhydric phenol on the electrode surface, thereby amplifying the electrocatalytic phenol signal. The fabricated MXene-based tyrosinase biosensor exhibited good analytical performance with a phenol detection range of 0.05 ~ 15.5 μmol L^−1^ and a detection limit of 12 nmol L^−1^.

Table 5 comprises the most recent reported MXene-based electrocatalytic sensors designed to detect various environmental pollutants. Although MXenes have shown promising potential in sensory devices, future investigations should be directed to anodic stabilization of MXene for the direct electrocatalytic determination of target pollutants.

**Table 5:** MXene-based electrocatalytic sensors designated to the detection of various environmental pollutant

MXene-based electrocatalyst	Pollutant	Technique	Linear Range (µM)	Detection limit (µM)	Reference
Nafion/ Ti_3_C_2_T_*x*_	Bromate ion	Differential pulse voltammetry	0.05–50	0.041	[81]
PANI-Ti_3_C_2_	Mercury ions	Anodic stripping voltammetry	5 × 10^–4^–1 × 10^–1^	8.5 × 10^–5^	[82]
AChE/Ag@ Ti_3_C_2_T_*x*_	Malathion	Differential pulse voltammetry	1 × 10^–8^–1 × 10^–2^	3.27 × 10^–9^	[83]
Ti_3_C_2_T_*x*_	Carbendazim	Differential pulse voltammetry	0.05–100	0.0103	[84]
Ti_3_C_2_T_*x*_ /ERGO	Carbendazim	Differential pulse voltammetry	0.002–10	0.00067	[85]
Ti_3_C_2_-MWCNT	CT HQ	Differential pulse voltammetry	2–150	0.0066 0.0039	[88]
Tyr-MXene-Chi	Phenol	Amperometric	0.05–15.5	0.012	[89]

*AChE* acetylcholinesterase, *ERGO* electrochemically reduced graphene oxide, *CT* catechol, *HQ* hydroquino

## Conclusions and perspective

Since their first discovery, MXenes, with their tunable surface chemistry, ease of functionalization, high hydrophilicity and excellent conductivity, have shown tremendous potential in multidisciplinary applications. In this review, we have outlined the recent progress of MXenes in environmental remediation applications, starting with adsorption and membrane separation, photocatalysis with their extension to electrocatalytic sensors designed particularly to detect toxins. The large surface area with the negatively charged surface has enabled MXene-based nanomaterials to exhibit promising adsorption abilities. MXene and related composites' sorption capabilities have been explored for diverse environmental applications ranging from adsorption and photocatalytic removal of toxic dyes, ions, and toxic compounds. Unlike conventional sorbents, e.g., carbon and graphene-based materials, MXenes offer ample advantages in tunable surface chemistry and high hydrophilicity, allowing easy surface-sorption of toxins.

Regarding adsorption and membrane separation, MXene sheets stability is yet a critical issue that directly influences the overall life-span of the MXene-based adsorbents. Moreover, the adsorption mechanism relies on the nature of the surface moieties. Thus, new synthetic routes and post-treatment methods could be anticipated to increase stability and induce selective sorption capability to MXene-based membranes. The use of MXenes in photocatalytic removal of pollutants requires efforts to engineer well-connected interfaces to improve photon adsorption and charge-carrier transportation. In this context, the engineered hybrids with improved interfacial arrangements and superior redox capability could produce efficient and broad-spectrum photocatalysts with robust degradation ability. The stability of MXenes is a bottle-neck issue for their application in environmental remediation technologies. The understanding of the oxidation pathways in different solvents and the subsequent lattice change of MXenes requires in-depth investigation. Moreover, the variation of the electronic structure of MXenes after doping with various metals and metal oxides to produce efficient photocatalysts should be focused on, for which the investigation approach on atomic level will be helpful.

In regards to the membrane, the control of water-flux is directly associated with MXenes flake size. Thus, both theoretical and experimental approaches should be adopted to realize an optimum flux rate with a high salt rejection rate besides revealing the underlying mechanism. The selective sorption/isolation of toxic species is another challenge for MXene-based adsorbents and membranes, which requires experimental procedures directed towards engineering MXene sheets with one type of uniform terminations. MXene-based membranes tend to swell, leading to the rejection of ions and low selectivity. Here, interlayer engineering to tune the d-spacing of MXene sheets can be a viable route to avoid swelling or structural collapse of MXene membranes.

Among the newly emerging applications, MXenes have gained progress in the area of electrocatalytic sensory systems. Unlike graphene, which offers good conductivity at the expense of low hydrophilicity, MXenes provide superior conductivity in addition to high dispersibility, recognizing them as an ideal platform for the electrocatalytic sensor. At present, the anodic stability of MXene is an issue that hinders the direct utilization of MXene for the electrocatalytic detection of pollutants. In this regard, the coupling of MXenes with electro-active polymer to construct composites have been proven effective in detecting toxins such as heavy metal ions. The use of MXenes has also been reported for enzyme-based sensors sensitive towards phenolic pollutants. Currently, the use of MXenes in the electrochemical sensor is still in infancy, requiring an in-depth understanding of its surface-related electrochemical behaviour. Although the integration of MXenes in electrocatalytic sensors has improved the signal sensitivity and the detection limit for various pollutants, the contribution of MXenes, in most cases, is merely limited to increase the conductivity.

In general, other MXene phases, i.e., V_4_C_3_T_*x*_ and Nb_2_CT_*x,*_ should also be investigated for their versatility and potential in environmental remediation technologies. Moreover, research should be directed to MXene derived hybrid composites that can contribute to the advancement of environmental remediation technologies.

## Data Availability

Not applicable.

## Abbreviations

PEI/PAA: Polyethylene polyimide /poly (acrylic acid)
MB: Methylene blue
ST: Safranine T
NR: Neutral red
MO: Methylene orange
RhB: Rhodamine B
DL-MXene: Delaminated MXene
PDDA: Poly (diallyl dimethylammonium chloride)
HTNs: Hierarchical titanate nanostructures
PVDF: Polyvinylidene difluoride
AAO: Anodic aluminum oxide
PC: Polycarbonate
PES: Polyethersulfone
PAN: Polyacrylonitrile
BGFO-20Sn: Gd and Sn co-doped bismuth ferrite BiFeO_3_ nanoparticles
BLFMO: La and Mn co-doped bismuth ferrite BiFeO_3_ nanoparticles
CR: Congo red
MB: Methyl blue
AB80: Acid blue 80
MO: Methyl orange
CBZ: Carbamazepine
RhB: Rhodamine B
SCP: Sulfachloropyridazine
SA: Salicylic acid
2,4-DNP: 2,4-Dinitrophenol
TC-H: Tetracycline hydrochloride
TPL: Thiamphenicol
CPL: Chloramphenicol
SMZ: Sulfamethazine
SFE: Sulfadimidine
CIP: Ciprofloxacin
4-NA: 4-Nitroaniline
AChE: Acetylcholinesterase
ERGO: Electrochemically reduced graphene oxide
CT: Catechol
HQ: Hydroquinone
2-NA: Nitroaniline
4-NP: 4-Nitrophenol

## References

[1] Shao L, Chen GQ (2013). Environ. Sci. Technol..

[2] Jasper JT, Yang Y, Hoffmann MR (2017). Environ. Sci. Technol..

[3] Robinson T, McMullan G, Marchant R, Nigam P (2001). Bioresour. Technol..

[4] Fu Y, Viraraghavan T (2001). Bioresour. Technol..

[5] Qu J (2008). J. Environ. Sci..

[6] Bagheri M, Mirbagheri SA (2018). Bioresour. Technol..

[7] Chong MN, Jin B, Chow CW, Saint C (2010). Water Res..

[8] Kempahanumakkagari S, Vellingiri K, Deep A, Kwon EE, Bolan N, Kim K-H (2018). Coord. Chem. Rev..

[9] Zhu Y, Murali S, Cai W, Li X, Suk JW, Potts JR, Ruoff RS (2010). Adv. Mater..

[10] Fanourakis SK, Bahamonde J, Bandara PC, Rodrigues DF (2020). NPJ Clean Water.

[11] Naguib M, Mashtalir O, Carle J, Presser VR, Lu J, Hultman L, Gogotsi Y, Barsoum M (2012). ACS Nano.

[12] Naguib M, Mochalin VN, Barsoum MW, Gogotsi Y (2014). Adv. Mater..

[13] Xu B, Gogotsi Y (2020). Adv. Funct. Mater..

[14] Zhu Q, Li J, Simon P, Xu B (2021). Energy Storage Mater..

[15] Xu B, Gogotsi Y (2020). Chin. Chem. Lett..

[16] Schultz T, Frey NC, Hantanasirisakul K, Park S, May SJ, Shenoy VB, Gogotsi Y, Koch N (2019). Chem. Mater..

[17] Li Z, Wu Y (2019). Small.

[18] Li J, Li X, Van der Bruggen B (2020). Environ. Sci. Nano.

[19] Mashtalir O, Cook KM, Mochalin VN, Crowe M, Barsoum MW, Gogotsi Y (2014). J. Mater. Chem. A.

[20] Wei Z, Peigen Z, Wubian T, Xia Q, Yamei Z, Ming S (2018). Mater. Chem. Phys..

[21] Li K, Zou G, Jiao T, Xing R, Zhang L, Zhou J, Zhang Q, Peng Q (2018). Colloids Surf. A Physicochem. Eng. Asp..

[22] Peng C, Wei P, Chen X, Zhang Y, Zhu F, Cao Y, Wang H, Yu H, Peng F (2018). Ceram. Int..

[23] Cai C, Wang R, Liu S, Yan X, Zhang L, Wang M, Tong Q, Jiao T (2020). Colloids Surf. A Physicochem. Eng. Asp..

[24] Carolin CF, Kumar PS, Saravanan A, Joshiba GJ, Naushad M (2017). J. Environ. Chem. Eng..

[25] Peng Q, Guo J, Zhang Q, Xiang J, Liu B, Zhou A, Liu R, Tian Y (2014). J. Am. Chem. Soc..

[26] Ying Y, Liu Y, Wang X, Mao Y, Cao W, Hu P, Peng X (2015). ACS Appl. Mater. Interfaces.

[27] Zou G, Guo J, Peng Q, Zhou A, Zhang Q, Liu B (2016). J. Mater. Chem. A.

[28] Fard AK, McKay G, Chamoun R, Rhadfi T, Preud'Homme H, Atieh MA (2017). J. Chem. Eng..

[29] Shahzad A, Rasool K, Miran W, Nawaz M, Jang J, Mahmoud KA, Lee DS (2017). ACS Sustain. Chem. Eng..

[30] Shahzad A, Rasool K, Miran W, Nawaz M, Jang J, Mahmoud KA, Lee DS (2018). J. Hazard. Mater..

[31] Zhang Q, Teng J, Zou G, Peng Q, Du Q, Jiao T, Xiang J (2016). Nanoscale.

[32] Pandey RP, Rasool K, Abdul Rasheed P, Mahmoud KA (2018). ACS Sustain. Chem. Eng..

[33] Rahman ROA, Ibrahium HA, Hung Y-T (2011). Water.

[34] Hwang SK, Kang S-M, Rethinasabapathy M, Roh C, Huh YS (2020). Chem. Eng. J..

[35] Wang L, Yuan L, Chen K, Zhang Y, Deng Q, Du S, Huang Q, Zheng L, Zhang J, Chai Z, Barsoum MW, Wang X, Shi W (2016). ACS Appl. Mater. Interfaces.

[36] Wang L, Tao W, Yuan L, Liu Z, Huang Q, Chai Z, Gibson JK, Shi W (2017). Chem. Comm..

[37] Wang L, Song H, Yuan L, Li Z, Zhang Y, Gibson JK, Zheng L, Chai Z, Shi W (2018). Environ. Sci. Technol..

[38] Wang L, Song H, Yuan L, Li Z, Zhang P, Gibson JK, Zheng L, Wang H, Chai Z, Shi W (2019). Environ. Sci. Technol..

[39] Li S, Wang L, Peng J, Zhai M, Shi W (2019). J. Chem. Eng..

[40] Zhang P, Wang L, Yuan L-Y, Lan J-H, Chai Z-F, Shi W-Q (2019). J. Chem. Eng..

[41] Goh PS, Ismail AF (2018). Desalination.

[42] Ren CE, Hatzell KB, Alhabeb M, Ling Z, Mahmoud KA, Gogotsi Y (2015). J. Phys. Chem. Lett..

[43] Ding L, Wei Y, Wang Y, Chen H, Caro J, Wang H (2017). Angew Chem. Int. Ed..

[44] Kang KM, Kim DW, Ren CE, Cho KM, Kim SJ, Choi JH, Nam YT, Gogotsi Y, Jung HT (2017). ACS Appl. Mater. Interfaces.

[45] Han R, Ma X, Xie Y, Teng D, Zhang S (2017). RSC Adv..

[46] Pandey RP, Rasool K, Madhavan VE, Aïssa B, Gogotsi Y, Mahmoud KA (2018). J. Mater. Chem. A.

[47] Gao X, Li Z-K, Xue J, Qian Y, Zhang L-Z, Caro J, Wang H (2019). J. Membr. Sci..

[48] Tran Thi Thuong H, Tran Thi Kim C, Nguyen Quang L, Kosslick H (2019). Pro. Nat. Sci.: Mater.

[49] Babu B, Akkinepally B, Shim J, Yoo K (2019). Ceram. Int..

[50] Jamble SN, Ghoderao KP, Kale RB (2018). Res. Chem. Intermed..

[51] Gao Y, Wang L, Zhou A, Li Z, Chen J, Bala H, Hu Q, Cao X (2015). Mater. Lett..

[52] Peng C, Yang X, Li Y, Yu H, Wang H, Peng F (2016). A.C.S. Appl. Mater. Interfaces.

[53] Peng C, Wang H, Yu H, Peng F (2017). Mater. Res. Bull..

[54] Shahzad A, Rasool K, Nawaz M, Miran W, Jang J, Moztahida M, Mahmoud KA, Lee DS (2018). J. Chem. Eng..

[55] Lu Y, Yao M, Zhou A, Hu Q, Wang L (2017). J. Nanomater..

[56] Wojciechowski T, Rozmyslowska-Wojciechowska A, Matyszczak G, Wrzecionek M, Olszyna A, Peter A, Mihaly-Cozmuta A, Nicula C, Mihaly-Cozmuta L, Podsiadlo S, Basiak D, Ziemkowska W, Jastrzebska A (2019). Inorg. Chem..

[57] Li J, Wang S, Du Y, Liao W (2018). Ceram. Int..

[58] Wang H, Wu Y, Xiao T, Yuan X, Zeng G, Tu W, Wu S, Lee HY, Tan YZ, Chew JW (2018). Appl. Catal. B Environ..

[59] Liu Q, Tan X, Wang S, Ma F, Znad H, Shen Z, Liu L, Liu S (2019). Environ. Sci. Nano.

[60] Zhou W, Zhu J, Wang F, Cao M, Zhao T (2017). Mater. Lett..

[61] Zhang H, Li M, Cao J, Tang Q, Kang P, Zhu C, Ma M (2018). Ceram. Int..

[62] Tariq A, Ali SI, Akinwande D, Rizwan S (2018). ACS omega.

[63] Iqbal MA, Irfan Ali S, Tariq A, Iqbal MZ, Rizwan S (2018). Reprints.

[64] Cao Y, Fang Y, Lei X, Tan B, Hu X, Liu B, Chen Q (2020). J. Hazard. Mater..

[65] Fang Y, Cao Y, Chen Q (2019). Ceram. Int..

[66] Cui C, Guo R, Xiao H, Ren E, Song Q, Xiang C, Lai X, Lan J, Jiang S (2020). Appl. Surf. Sci..

[67] Cai T, Wang L, Liu Y, Zhang S, Dong W, Chen H, Yi X, Yuan J, Xia X, Liu C, Luo S (2018). Appl. Catal. B Environ..

[68] Liu N, Lu N, Su Y, Wang P, Quan X (2019). Sep. Purif. Technol..

[69] Ding X, Li C, Wang L, Feng L, Han D, Wang W (2019). Mater. Lett..

[70] Xie X, Zhang N (2020). Adv. Funct. Mater..

[71] Zhu Y, Wu Z, Xie X, Zhang N (2020). Pure Appl. Chem..

[72] Song F, Li G, Zhu Y, Wu Z, Xie X, Zhang N (2020). J. Mater. Chem. A.

[73] Chen Y, Xie X, Xin X, Tang ZR, Xu YJ (2019). ACS Nano.

[74] Shi X, Wang P, Lan L, Jia S, Wei Z (2019). J. Mater. Sci. Mater. Electron..

[75] Li K, Jiao T, Xing R, Zou G, Zhao Q, Zhou J, Zhang L, Peng Q (2018). Green Energy Environ..

[76] Xie X, Wu Z, Zhang N (2020). Chin. Chem. Lett..

[77] Luo S, Wang R, Yin J, Jiao T, Chen K, Zou G, Zhang L, Zhou J, Zhang L, Peng Q (2019). ACS Omega.

[78] Liu Y, Luo R, Li Y, Qi J, Wang C, Li J, Sun X, Wang L (2018). J. Chem. Eng..

[79] Yin J, Zhang L, Jiao T, Zou G, Bai Z, Chen Y, Zhang Q, Xia M, Peng Q (2019). Nanomaterials.

[80] Soomro RA, Jawaid S, Zhu Q, Abbas Z, Xu B (2020). Chin. Chem. Lett..

[81] Rasheed PA, Pandey RP, Rasool K, Mahmoud KA (2018). Sens. Actuators B Chem..

[82] Cheng H (2020). Int. J. Electrochem. Sci..

[83] Jiang Y, Zhang X, Pei L, Yue S, Ma L, Zhou L, Huang Z, He Y, Gao J (2018). J. Chem. Eng..

[84] Wu D, Wu M, Yang J, Zhang H, Xie K, Lin C, Yu A, Yu J, Fu L (2018). Mater. Lett..

[85] Xie Y, Gao F, Tu X, Ma X, Xu Q, Dai R, Huang X, Yu Y, Lu AL (2019). J. Electrochem. Soc..

[86] Soomro R, Jawaid S, Kalawar N, Tunesi M, Karakus S, Kilislioglu A, Willander M (2020). Biosens. Bioelectron..

[87] Kalambate PK, Gadhari NS, Li X, Rao Z, Navale ST, Shen Y, Patil VR, Huang Y (2019). Trends Anal. Chem..

[88] Huang R, Chen S, Yu J, Jiang X (2019). Ecotoxicol. Environ. Saf..

[89] Wu L, Lu X, Dhanjai, Wu ZS, Dong Y, Wang X, Zheng S, Chen J (2018). Biosens. Bioelectron..

